# Molecular docking analysis and evaluation of the antimicrobial properties of the constituents of *Geranium wallichianum* D. Don ex Sweet from Kashmir Himalaya

**DOI:** 10.1038/s41598-022-16102-9

**Published:** 2022-07-22

**Authors:** Wajahat Rashid Mir, Basharat Ahmad Bhat, Muzafar Ahmad Rather, Showkeen Muzamil, Abdullah Almilaibary, Mustfa Alkhanani, Manzoor Ahmad Mir

**Affiliations:** 1 Department of Bioresources, School of Biological Sciences, University of Kashmir, Srinagar, J&K 190006 India; 2 Molecular Biology Laboratory, Faculty of Veterinary Sciences and Animal Husbandry, SKUAST-K, Srinagar, India; 3 Department of Family and Community Medicine, Faculty of Medicine, Albaha University, Albaha, 65511 Kingdom of Saudi Arabia; 4 Biology Department, College of Sciences, University of Hafr Al Batin, Hafar Al Batin, 31991 Kingdom of Saudi Arabia

**Keywords:** Biochemistry, Biological techniques, Cell biology, Computational biology and bioinformatics, Microbiology

## Abstract

*Geranium wallichianum* D. Don ex Sweet is a well-known medicinal plant in Kashmir Himalya. The evidence for its modern medicinal applications remains majorly unexplored. The present study was undertaken to elucidate the detailed antimicrobial promises of different crude extracts (methanolic, ethanolic, petroleum ether, and ethyl acetate) of *G. wallichainum* against common human bacterial and fungal pathogens in order to scientifically validate its traditional use. The LC–MS analysis of *G. wallichainum* yielded 141 bioactive compounds with the vast majority of them having therapeutic applications. Determination of minimum inhibitory concentrations (MICs) by broth microdilution method of *G. wallichainum* was tested against bacterial and fungal pathogens with MICs ranging from 0.39 to 400 µg/mL. Furthermore, virtual ligands screening yielded elatine, kaempferol, and germacrene-A as medicinally most active constituents and the potential inhibitors of penicillin-binding protein (PBP), dihydropteroate synthase (DHPS), elongation factor-Tu (Eu-Tu), ABC transporter, 1,3 beta glycan, and beta-tubulin. The root mean square deviation (RMSD) graphs obtained through the molecular dynamic simulations (MDS) indicated the true bonding interactions which were further validated using root mean square fluctuation (RMSF) graphs which provided a better understanding of the amino acids present in the proteins responsible for the molecular motions and fluctuations. The effective binding of elatine, kaempferol, and germacrene-A with these proteins provides ground for further research to understand the underlying mechanism that ceases the growth of these microbes.

## Introduction

Antibiotics are crucial weapons in fighting various microbial infections and have significantly improved human health since their introduction^1,2^. However, the last few years have witnessed the excessive use of antibiotics cause resistance, leading to hazardous effects on human health^2,3^. Researchers are trying to develop new drugs with no resistance. As a result, the traditional systems of medicine are gaining enormous popularity since they are more natural, environmentally friendly, and devoid of adverse effects^4,5^. Thus, despite the numerous advantages of current synthetic medicines, people continue to choose plant-based natural remedies over synthetic medications^6–9^. The majority of medicinal plants are unique in their potential to treat and cure various human health problems, owing to several essential phytoconstituents in different plant parts^10^. Numerous bioactive chemicals found in medicinal plants have pharmacological activities like antimicrobial, anticancer, antioxidant and anti-inflammation properties^6,10–14^. Although many plant species have many biological metabolites, only a limited number have been investigated and confirmed to represent a substantial source of natural compounds. It is essential to create good screening processes to discover new compounds^15^. The extraction and characterization of a large number of these bioactive chemicals from various medicinal plants have resulted in the administration of specific medications with a high activity profile^16,17^. The first screening of medicinal plants using chromatographic and spectrometric methods offers essential information on their chemical and pharmacological characteristics, which help to select biologically active plants^18^.

Liquid chromatography-mass spectrometry (LC–MS) has been mainly used in recent years to detect functional groups and identify a variety of bioactive therapeutic phytocompounds present in medicinal plants^19,20^. LC–MS is one of the most effective, rapid, and precise method for detecting a wide variety of chemicals, including alkaloids, nitro compounds, asters, alcohols, organic acids, steroids, long-chain hydrocarbons and amino acids^21^, and utilises a little amount of extracts of plants.

Computer-aided approaches for drug discovery have evolved as improved technologies that can be used to screen for medications derived from phytochemicals present in a variety of medicinal plants. Computational prediction models are critical in guiding the methodology selection process for pharmaceutical and technology research. They have also been used in in silico forecast of pharmacokinetic, pharmacological and toxicological performance^22^. Presently, molecular docking is an efficient and cost-effective strategy for developing and testing pharmaceuticals. This approach generates data on drug and receptor interactions that may be used to predict the orientation of drug candidates when bound to their target protein^23^. Additionally, this technique facilitates systemic investigation by non-covalently placing a molecule into the binding site of an object macromolecule, resulting in specific binding at the active sites of every ligand^24–26^. In this aspect, the present study used the LC–MS method to detect and identify phytochemical components contained in the medicinal plant.

*Geranium wallichianum* D. Don ex Sweet is a species belonging to the Geraniaceae family^27^. *Geranium* L. is a large genus with 325 species found worldwide except in lowland tropical climates^27^. In India, 27 *Geranium* species have been recorded, with the most remarkable diversity occurring in the nation's temperate Himalaya and tropical mountainous areas, particularly the Deccan peninsula, Western Ghats, and the northeast region. It is mainly found in high altitude Himalayas of Jammu and Kashmir^28^.

Polyphenol rich extracts of *Geranium* L. species as potential natural antioxidant and antimicrobial agents^29^. *Geranium wallichianum* D. Don ex Sweet is a well-known traditional plant used by herbalists to treat backache, sexual debility, joint pain, colic, jaundice, and kidney and spleen disorder^30^. *G. wallichianum* is usually used as tonic by women especially for physical fitness and other internal body complaints^31^. In different assay the crude extracts and different fractions of rhizomes and leaves showed varied degree of antimicrobial activities and enzyme inhibitions^27,32^. It is also rich in phytochemicals such as ursolic acid, β-sitosterol, stigmasterol, β-sitosterolgalactoside herniarin, and 2, 4, 6-trihydroxyethylbenzoate^33^. Ursolic acid (UA) is a natural terpene compound exhibiting many pharmaceutical properties^34^. However, there is a significant gap between the paucity of scientific research on *G. wallichianum* and its value in traditional medicine. So, more research is needed to determine the potential therapeutic efficacy and possible mechanisms of action of *G. wallichianum*. In response to all of the above, the current study was designed to (1) evaluate the *in-vitro* antimicrobial activity of the different polarity extracts of *G. wallichianum* roots, including petroleum ether, ethyl acetate, methanol and ethanol (2) identify the potential bioactive components present in the active extract through the LC/MS technique; and (3) apply an *in-silico* analysis for the most abundant compounds against the target proteins involved in the life cycle of bacteria and fungi.

## Methodology

### Collection of plant sample

The plant material of *G. wallichianum* was collected from different sites of Kashmir valley. The material was recognized and confirmed by Akhtar H. Malik, before its drying in the shade. Voucher specimen number (2954) was kept in the Department of Taxonomy at University of Kashmir (Table 1). The permission for collection of plant material was taken from the concerned authorities.

**Table 1:** Details of the locations where plant samples of *G. wallichianum* were collected from four main sites in India's Jammu and Kashmir union region for ethnopharmacological investigation.

Name of sampling site	Geographical coordinates			Sample collection date	Sample type
	Latitude	Longitude	Altitude (amsl)		
Sadhna Pass	34.4016°N	73.9535°E	3000	23-07-20	Whole plant
Kupwara	34°31′33″N	74°15′19″E	3545	26-07-20	Whole plant
Sinthantop	33.5811°N	75.5102°E	3784	02-08-20	Whole plant
Daksum (Anantnag)	33.6114°N	75.4359°E	2438	09-08-20	Whole plant
Uri (Baramullah)	34.0881°N	74.0340°E	1579	04-08-20	Whole plant

### Plant root extract preparation

Petroleum ether, ethyl acetate, ethanol and methanol were chosen as extraction solvents based on their polarity of index. A mechanical grinder is used to powder about 800 g of the roots of the *G. wallichianum* plant washed with deionized water, shade dried for 10–15 days, pulverized with a mechanical grinder, and stored in an airtight container. Furthermore, 200 g of *G. wallichianum* powder is mixed in 20 millilitres of Milli Q water and placed for 15 min in a water bath at 55 °C. To obtain the plant extract using the Soxhlet apparatus method, petroleum ether, ethyl acetate, ethyl alcohol, and methyl alcohol solvents were chosen for their polarity index. The extracts were filtered using Whatman No. 1 filter paper, and the extracts were then concentrated using a rotating vacuum evaporator, which were  then stored at 4 °C for future purposes^4^.

### Liquid chromatography and mass spectrometry analysis

The LC–MS analysis was specifically carried out using a Nexera UHPLC with quaternary pump, Autosampler, conspicuous degassing unit and DAD unit. It assures high fecundity, increased output, enhanced accuracy, and better results. The solvents used were methanol, ethyl alcohol, ethyl acetate, and petroleum ether, which were eluted at 1 ml/min. All of the solutions were passed through 0.45 mu nylon sheets following ultrasonication. The chromatograms were inspected at 270 nm, and the results were collected using lab-developed software^35^.

### Microbial strains and culture

Council of Scientific and Industrial Research, Institute of Microbial Technology (CSIR-IMTech) in Chandigarh, Punjab India furnished the microbial strains for the study. Six of the nine microbial species designated for the experiment were bacterium strains, while three were fungal strains that were evaluated for antifungal activity. *Escherchia coli, Mycobacterium luteus, Streptococcus pneumoniae, Klebsiella pneumoniae, Neisseria mucosa, Haemophilus influenzae, Candida albicans, Candida paropsilosis* and *Candida glabrata* were used for the current research. Subcultures of bacterial strains were performed on Muller Hinton Agar [MHA] media. They were grown for 24 h at 37 °C on an agar medium until visible colonies emerged on the plate. Subcultures of fungal strains were grown in YPED broth on agar medium until sporulation occurred, which typically took 5 days. Bacteria colonies and spores of fungal strains were obtained in Muller Hinton Agar [MHA] and YEPD Broth, respectively, until the late log growth phase. The bacterial and fungal strains were kept at −70 °C in 1 ml glycerol stocks.

### Minimum inhibitory concentration (MIC) through broth dilution method

Minimum Inhibitory Concentration (MIC) of various plant extracts of *G. wallichianum* were measured using the Micro Dilution Method with slight modifications in 96 well plates (Corning; polystyrene; Flat Bottom). The different plant extracts concentrations ranged from .39 to 400 μg/ml, and ciprofloxacin was taken as a positive antibacterial agent (0.039–20 μg/ml). 50 μl of exponentially grown bacterial cultures were inoculated on plates, and the final volume was maintained as 200 μl. In 96 well plates, drug-free growth and drug-free medium control are included. The MIC values of plates were measured after 24 h of incubation at 37 °C. MIC was referred to as the lowest antimicrobial concentrations capable of suppressing detectable bacterial growth. Clinical and Laboratory Standards Institute (CLSI) criteria was used to determine the antifungal activity of plant extracts. Amphotericin-B was taken as a positive antifungal agent. MIC reading for antifungal activity was measured after 24, 48 and 72 h of incubation at 35 °C^36^.

### Protein preparation

**Figure 1:**
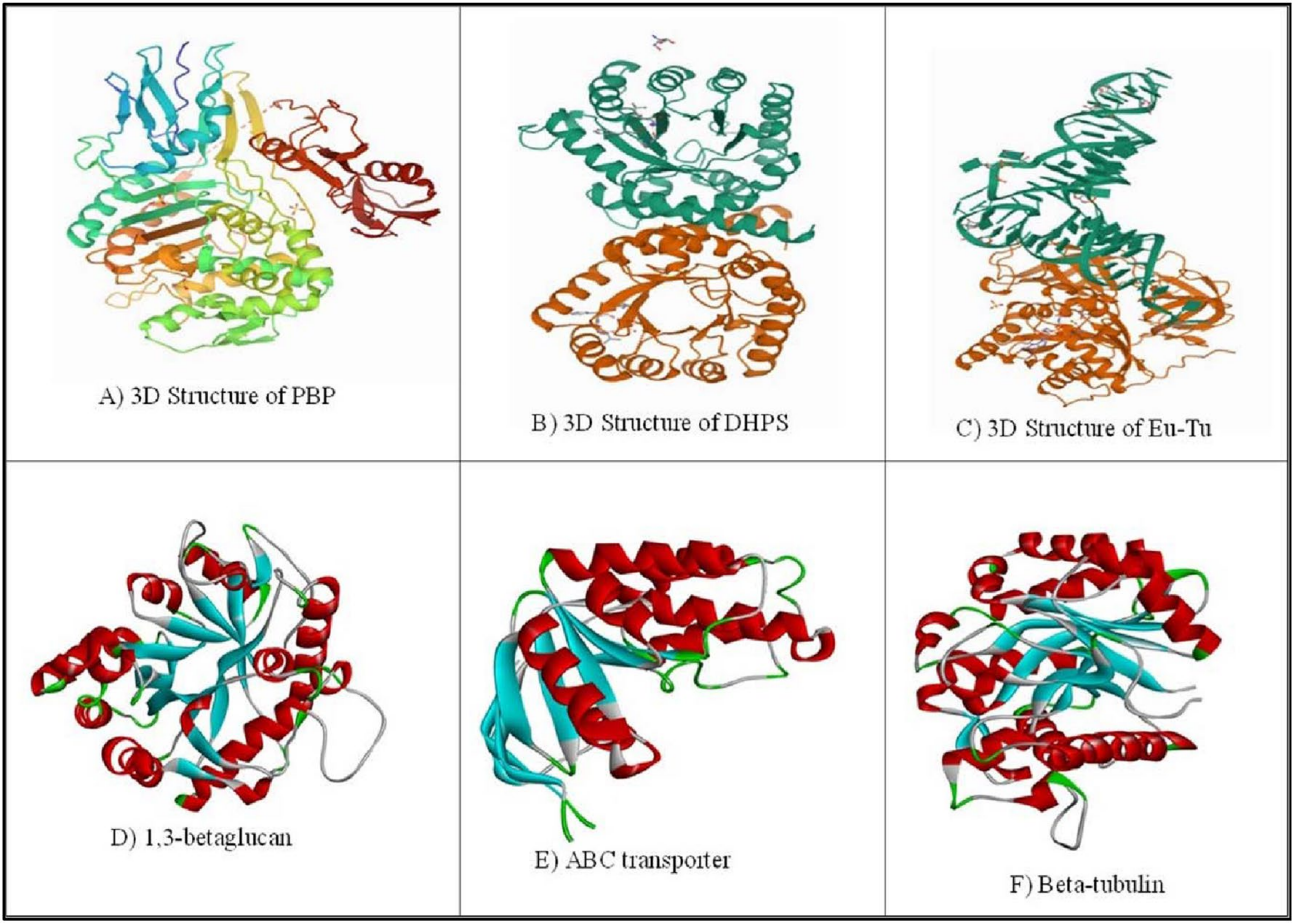
3D structure of different microbial target proteins.

In the present study, we have selected various bacterial and fungal target proteins such as penicillin-binding protein (PBP), dihydropteroate synthase (DHPS), elongation factor-Tu (Eu-Tu), ABC transporter, 1,3 β-glycan and beta-tubulin. All six proteins play an important role in the life cycle of bacteria and fungi. Based on the role performed by these proteins, we have selected these protein targets. The targeted macromolecules such as penicillin-binding protein, dihydropteroate synthase, elongation factor-Tu, beta-tubulin, ABC transporter and 1,3-Betaglycan were obtained from the RCSB PDB database^37^ and extracted as a PDB file (Fig. 1). After that, these biomolecules were individually entered into the molecular docking software AutoDock^25^. To begin, the proteins were further trimmed by extracting the cocrystallized ligands using Biovia Software. The protein was then processed by eliminating water molecules, removing superfluous chains or heteroatoms, introducing hydrogen, estimating charges (Kollman charges), and converting it to a pdbqt file. Ultimately, the cocrystallized ligands were placed in the centre of the grid box. Possible active sites of each target were determined using CASTp web server^38^. For docking with Autodock Vina^25^, the grid box measurements were recorded in a config.txt format. The co-crystallized ligands were then deleted from the resulting protein pdbqt files.

### Ligand preparation

The bioactive ligand molecules, Elatine, Kaempferol, and Germacrene A, were downloaded from the PubChem^39^ directory as 3D Standard Data Format (3D SDF) format. PyMol^40^ was used to translate the ligands from 3D SDF files to Protein Data Bank (PDB) format. These ligand molecules were independently uploaded into the AutoDock Tools during ligand preparation. Gasteiger charges were introduced to the compounds. Additionally, non-polar hydrogen atoms were combined, and rotational interactions were identified and altered.

### Purification and refinement of proteins and ligands

Unwanted interactions, disparate bindings, ligand compounds, molecules of water, and other impurities were removed from the macromolecule using Dassault Systems Biovia Discovery Studio Visualizer. To facilitate better interactions, only polar hydrogens were introduced to the protein during the preparation, following the addition of Kollman charges. After protein fabrication, all peptides and motifs were evaluated in Discovery studio for effectual active binding site prediction and saved in pdb format. The 3-dimensional and 2-dimensional structures of the ligands Elatine, Kaempferol, and Germacren A, were obtained using the PubChem^39^ repository. The 3D structures of Microbial Target Proteins were extracted from the RCSB PDB^37^ libraries and downloaded as pdf files.

### Molecular docking analysis

The molecular docking analysis of all the selected phytocompounds were subjected to AutoDock Vina 4.0^25^ using the script standard method. Both target proteins and selected compounds were then saved in pdbqt format after combining non-polar hydrogens. Molecular docking was performed within a grid box dimension 22 × 26 × 21 Å. It was necessary to design grid boxes with particular dimensions and 0.3 Å spacing. Docking studies of the protein–ligand complex were carried out in accordance with the Lamarckian Genetic Algorithm (LGA)^40^. All the binding affinities were measured and considered for dynamic simulation studies. Biovia Discovery studio^40^ was used to investigate the interacted amino acids and docked poses of the complex structures.

### Molecular dynamic simulation

The docking calculation were performed by Desmond Schrodinger v3.8^41^ with the best binding affinity compound. The current study utilized the NPT ensemble with the 300 K temperature and 1 bar pressure in all runs for nanoseconds. During dynamic simulation, the OPLS_2005 force field was employed for the hit compound, followed by the electrostatic charges were analyzed through the Ewald method. All the possible trajectories were considered at 4.0 picosecond intervals for better accuracy. Ligand and protein behaviour were analyzed using the simulation interaction tool implemented in the Desmond package tool^41^ as well as the stability of the complex was monitored by showing the Root Mean Square of Deviation (RMSD) and Root Mean Square of Deviation (RMSF) of the complex.

### Plant material collection statement

The permission for collection of plant material (*G. wallichainum* D. Don ex sweet) was taken from the concerned authorities. Further, all local, national or international guidelines and legislation were adhered to in the production of this study.

## Results

### Preliminary phytochemical screening

The phytochemical study of various extracts from the roots of *G. wallichianum* revealed a variety of phytochemicals, such as flavonoids, phenolics, terpenoids, saponins and tannins present in the plant extracts as represented in Table 2.

**Table 2:** Results of preliminary tests of *G. wallichianum.*

Tests	Inference	Methanol	Ethanol	Ethyl acetate	Petroleum ether
**Carbohydrates**					
Molisch’s test	Violet ring	+	+	+	+
Fehling’s test	Formation of yellow pot	+	−	+	−
Benedict’s test	Red precipitate	+	+	+	+
**Anthraquinone glycosides**					
Anthraquinone glycosides	The ammoniacal layer turns pink	−	+	+	+
**Saponin glycosides**					
Foam test	Persistent foam	+	+	+	+
**Flavonoids**					
Shinoda test	Pink color appears	+	+	+	+
Alkaline Reagent test	Concentrated yellow color	+	−	+	+
**Tannins and phenolics**					
FeCl_3_ test	Black color	+	+	+	+
Lead acetate test	White precipitate	+	+	+	−
**Steroids**					
Salkowski reaction	Chloroform layer appears red	+	−	+	+
**Alkaloids**					
Mayer’s test	Formation of precipitate	−	−	+	+
Dragendroff’s test	Organic precipitate	+	+	+	+
Wagner’s test	Formation of radish brown precipitate	+	+	−	−
**Terpenoids**					
Terpenoid test	Grey color	+	+	+	+

### Liquid chromatography-mass spectrometry (LC–MS) analysis of plant extracts

**Figure 2:**
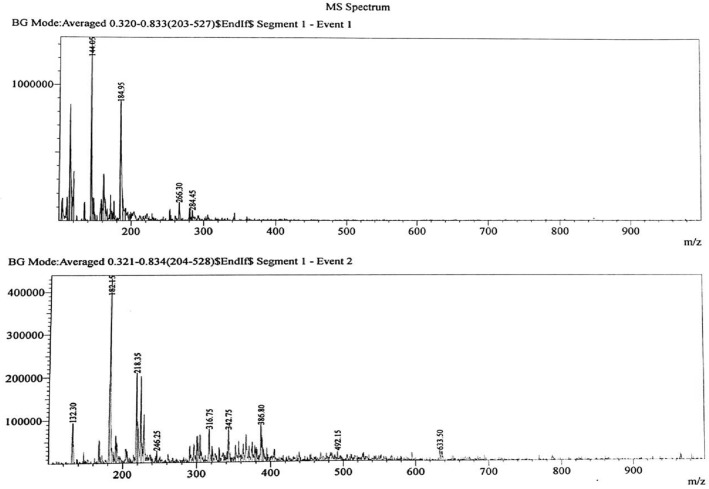
LC–MS-ESI–MS chromatograms of reference compounds using Nexera in Methanolic extract.

**Figure 3:**
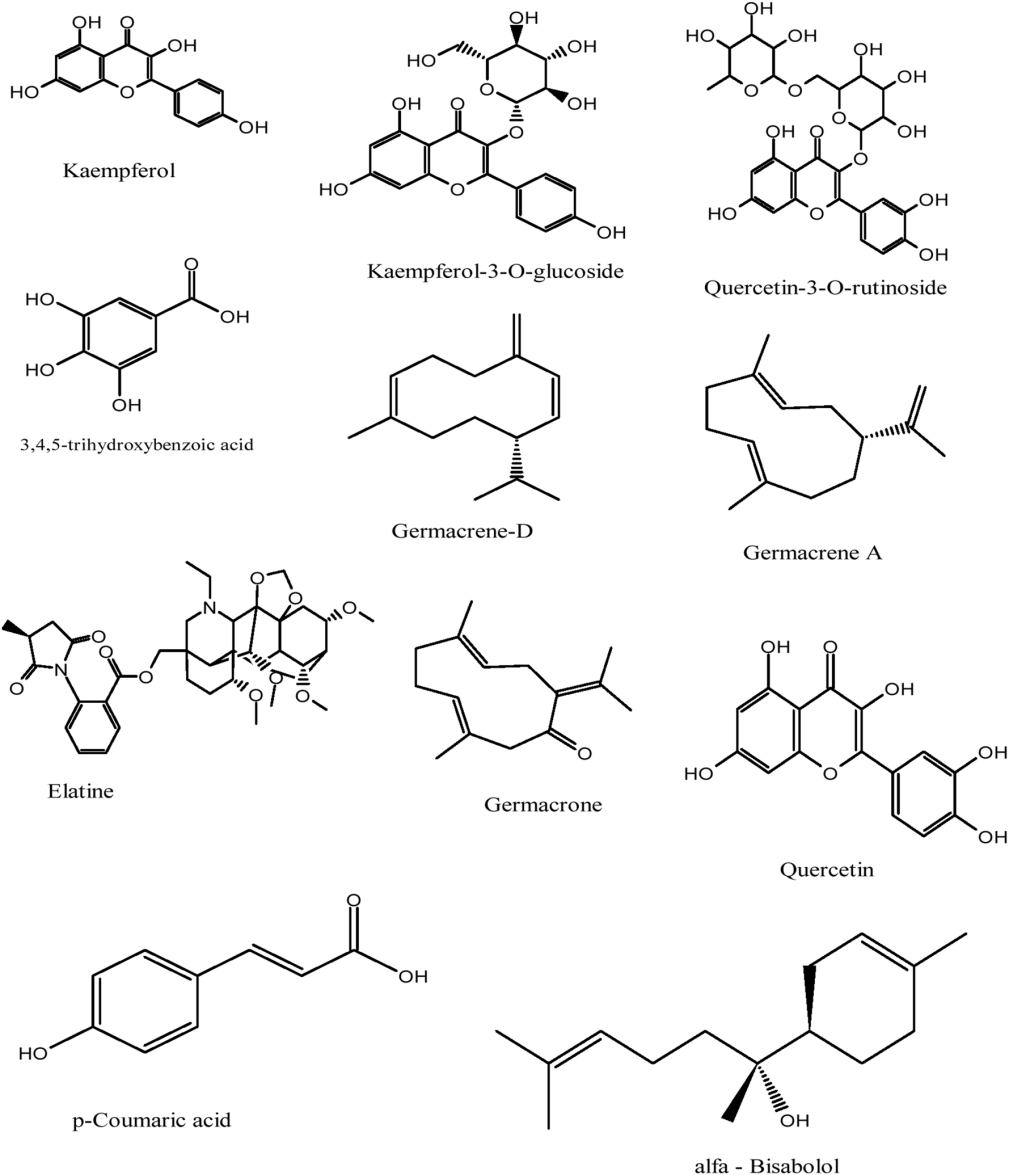
Structures of the compounds identified based on LC–MS from *G. wallichainum.*

Quantitative and qualitative analysis of the various extracts of *G. wallichianum* were determined by LC–MS. The LC–MS total ion chromatograms of various *G. wallichianum* extracts are shown in (Fig. 2). The solvent extraction was carried out by Soxhlet extraction and was subjected to LC–MS analysis to obtain 141 important bioactive phytocompounds through in-depth research. Some of the important ones are shown in (Table 3, Fig. 3). The maximum number of phytocompounds were obtained using the methanolic extract (40) followed by ethyl acetate (36), ethanol (33) and petroleum ether extracts (32) (Supplementary file). The identified compounds belong to the various secondary metabolites like terpenoids, alkaloids, aliphatic compounds, and phenolics. Some of the examples are Kaempferol, Quercetin, Kaempferol-3-O-glucoside, Quercetin-3-O-rutinoside, Gallic acid, Germacrene-D, Germacrene A, Elatine, Germacrone, α- Bisabolol and p-Coumaric acid (Table 3).

**Table 3:** The major components found in *G. wallichianum* based on LC–MS analysis.

Compound name	IUPAC name	Molecular formula	Molecular weight (g/mol)
Kaempferol	3,5,7-Trihydroxy-2-(4-hydroxyphenyl) chromen-4-one	C_15_H_10_O_6_	286.24
Kaempferol-3-O-glucoside	5,7-Dihydroxy-2-(4-hydroxyphenyl)-3-[(2S,3R,4S,5S,6R)-3,4,5-trihydroxy-6-(hydroxymethyl) oxan-2-yl] oxychromen-4-one	C_21_H_20_O_11_	448.4
Quercetin-3-O rutinoside	2-(3,4-Dihydroxyphenyl)-5,7-dihydroxy-3-[3,4,5-trihydroxy-6-[(3,4,5-trihydroxy-6-methyloxan-2-yl) oxymethyl] oxan-2-yl] oxychromen-4-one	C_27_H_30_O_16_	610.5
Gallic acid	3,4,5-Trihydroxybenzoic acid	C_7_H_6_O_5_	170.12
Germacrene D	(1Z,6Z,8S)-1-methyl-5-methylidene-8-propan-2-ylcyclodeca-1,6-diene	C_15_H_24_	204.35
Germacrene A	(1E,5E,8R)-1,5-dimethyl-8-prop-1-en-2-ylcyclodeca-1,5-diene	C_15_H_24_	204.35
Elatine	[(4S,6S,19R,21R)-14-ethyl-4,6,19,21-tetramethoxy-9,11-dioxa-14 azaheptacyclo [10.7.2.12,5.01,13.03,8.08,12.016,20] docosan-16-yl] methyl 2-[(3S)-3-methyl-2,5-dioxopyrrolidin-1-yl] benzoate	C_38_H_50_N_2_O_10_	694.8
Germacrone	(3E,7E)-3,7-dimethyl-10-propan-2-ylidenecyclodeca-3,7-dien-1-one	C_15_H_22_O	218.33
Quercetin	2-(3,4-Dihydroxyphenyl)-3,5,7-trihydroxychromen-4-one	C_15_H_10_O_7_	302.23
p- Coumaric acid	(2R)-6-methyl-2-[(1R)-4-methylcyclohex-3-en-1-yl] hept-5-en-2-ol	C_15_H_26_O	222.37
Alfa Bisabolol	(E)-3-(4-hydroxyphenyl) prop-2-enoic acid	C_9_H_8_O_3_	164.16

### Antimicrobial activity

Research studies have revealed that *G. wallichianum* possess antimicrobial potential against various strains of bacteria and fungi^32^. Regardless of the conducted research, it is complicated to compare the results, mainly due to differences in composition and origin of the plant, employed extraction techniques, the concentration of the obtained extracts, tested microorganisms, and so forth. Toward this end, it was necessary to evaluate the antimicrobial properties of the *G. wallichainum* dry extracts (ethyl acetate, Petroleum ether, ethanol and methanol) obtained in the present study. The minimum inhibitory concentration (MIC) was determined for all tested microorganisms. The antimicrobial activity of various extracts of *G. wallichianum* had showed strong antimicrobial potential against the selected microorganisms. MICs of standard antimicrobial drug targets such as ciprofloxacin and amphotericin B through broth dilution are shown in Table 4. Ethyl acetate had showed strong antimicrobial activity as compared to all other extracts. The MIC values of ethyl acetate extracts of *G. wallichianum* against *M. luteus, H. influenzae, S. pneumonia, K. pneumoniae, N. mucosa* and *E. coli* were 3.12, 6.25, 12.5, 25, 25, and 100 μg/mL, respectively*.* Plant extracts had shown less antimicrobial activity against the fungal strains viz, *C. albicans, C. glabrata and C. paropsilosis* compared to the bacterial strains. The antimicrobial potential of *G. wallichianum* extracts against selected bacterial and fungal strains observed by the MIC method is presented in Table 4.

**Table 4:** *Invitro* antimicrobial activity of different extracts of *G. wallichianum.*

Strain		MIC (µg/mL)*			
	ME	ET	EA	PE	CIP/AMF-B
*E. coli* (MTCC 443)	100	50	100	100	0.625
*M. luteus* (10240)	6.25	3.12	3.12	1.56	1.25
*K. pneumoniae* (MTCC 19)	6.25	25	25	25	0.039
*S. pneumoniae* (MTCC 655)	25	25	12.5	12.5	0.625
*H. influenzae* (MTCC 3826)	25	25	6.25	25	1.25
*N. mucosa* (MTCC 1772)	25	25	25	25	03.12
*C. albicans* (ATCC 24433)	200	6.25	> 400	> 400	1.25
*C. glabrata* (ATCC2001)	> 400	> 400	> 400	> 400	2 .5
*C. Paropsilosis* (ATCCC90018)	> 400	> 400	> 400	> 400	2 .5

*Results are the average of the triplicate readings. Where *CIP:* Ciprofloxacin (Positive antibacterial agent), *AMF-B:* Amphotericin-B (Positive antifungal agent), *PE:* Petroleum Ether, *ET:* Ethanol, *ME:* Methanol, *EA:* Ethyl Acetate.

### Molecular docking analysis

**Figure 4:**
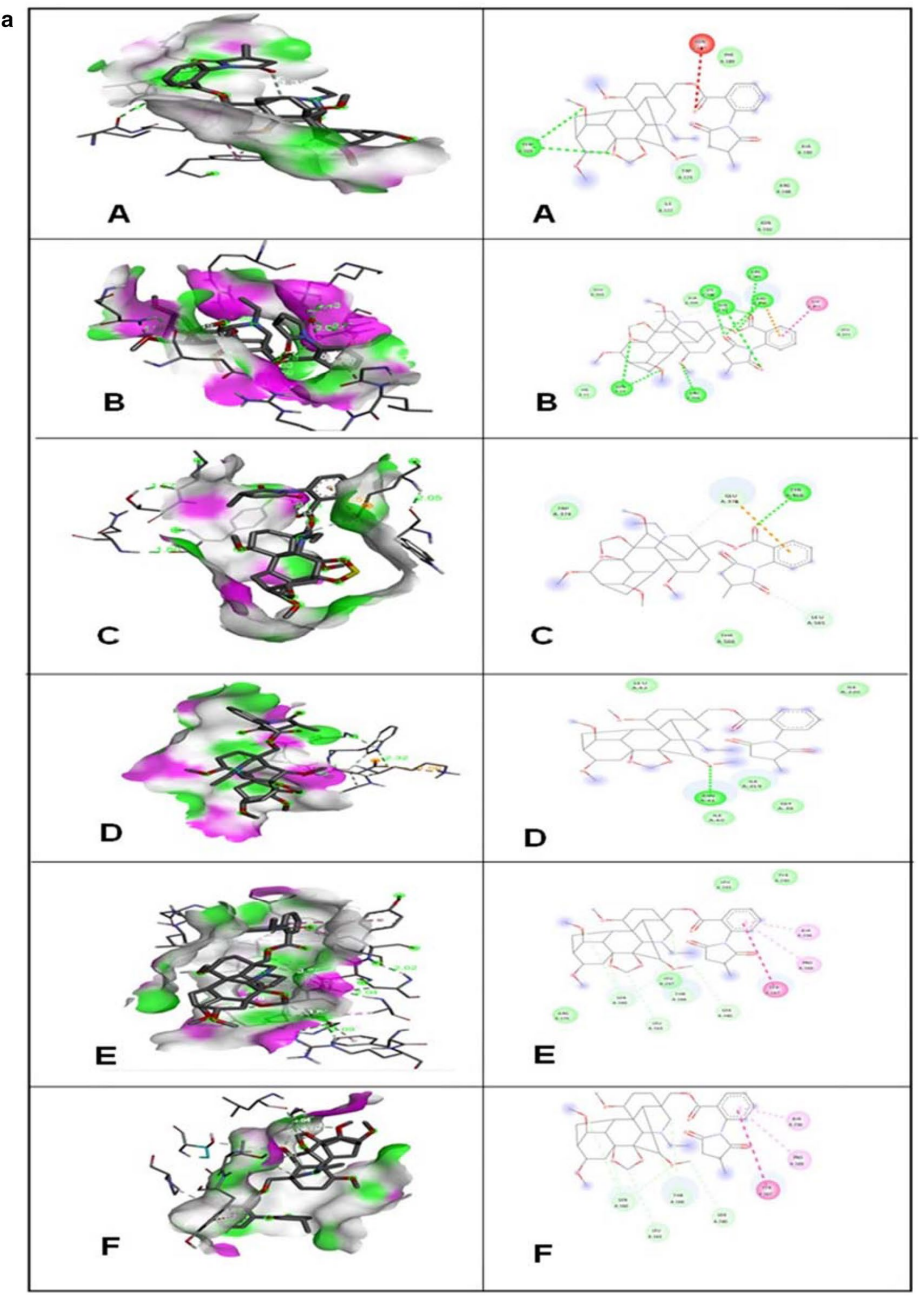

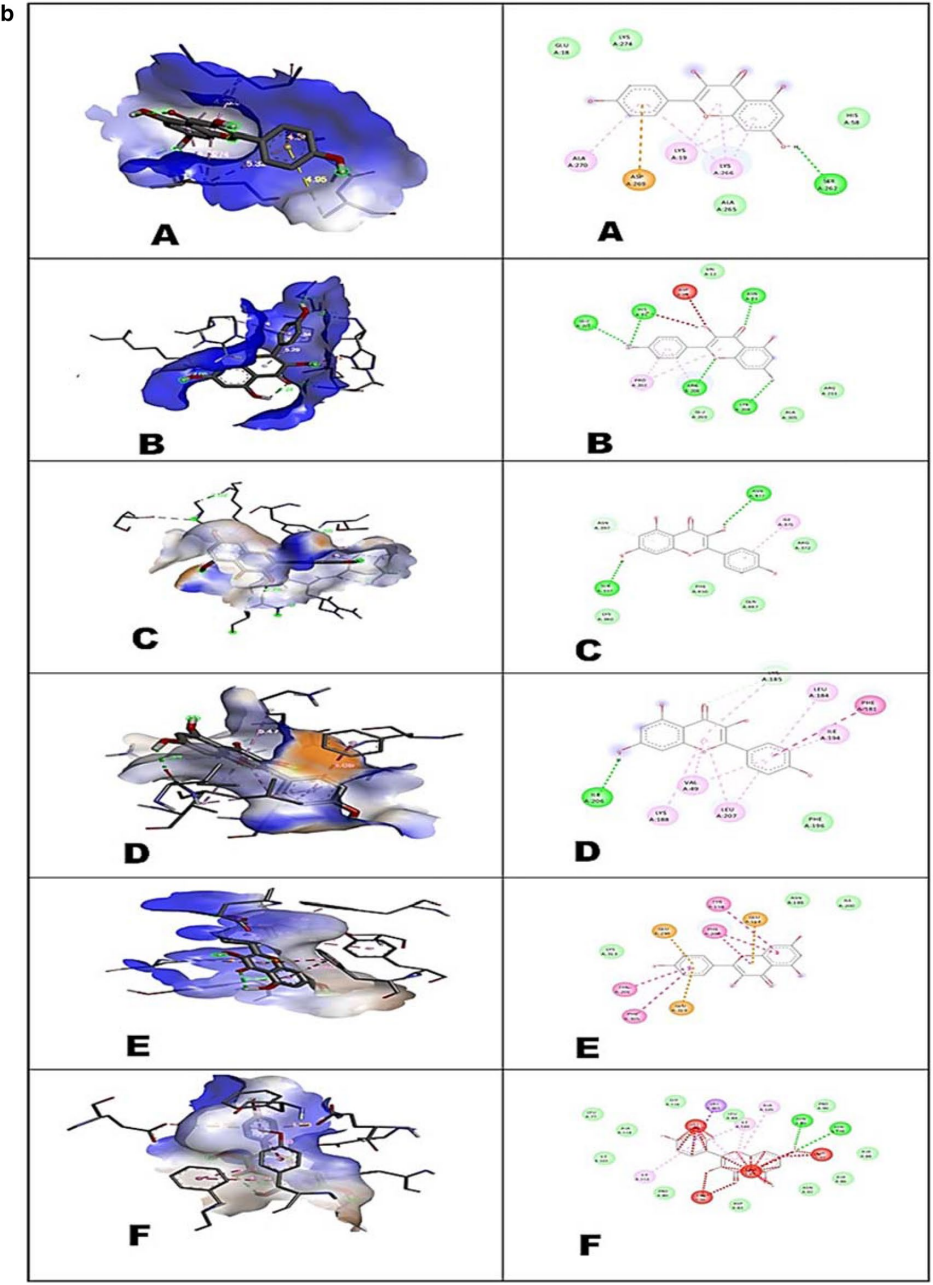

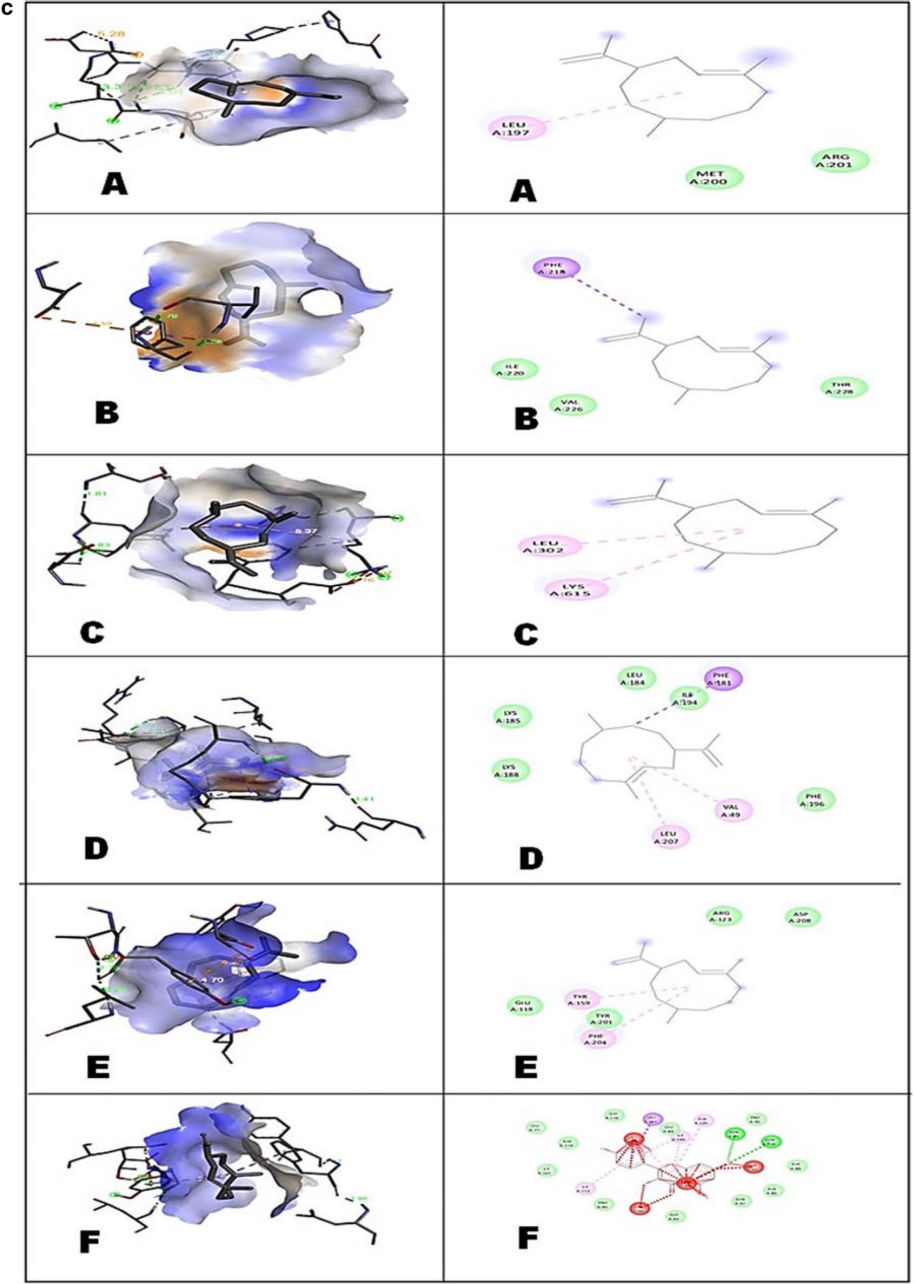
(**a**) 3D interactions of Ligands with (A) Dihydropteroate synthase (B) Elongation factor Tu and (C) Penicillin Binding Protein (D) ABC transporter (E) 1,3-Betaglycan (F) Beta-tubulin with Elatine and 2D structure of ligands interacted with respective amino acids. Read the text for further information. (**b**) 3D interactions of Ligands with (A) Dihydropteroate synthase (B) Elongation factor Tu and (C) Penicillin Binding Protein (D) ABC transporter (E) 1,3-Betaglycan (F) Beta-tubulin with Kaempherol and 2D structure of ligands interacted with respective amino acids. Read the text for further information. (**c**). 3D interactions of Ligands with (A) Dihydropteroate synthase (B) Elongation factor Tu and (C) Penicillin Binding Protein (D) ABC transporter (E) 1,3-Betaglycan (F) Beta-tubulin with Germacrene A and 2D structure of ligands interacted with respective amino acids. Read the text for further information.

In docking results, the binding affinity (Docking Free energy) and amino acid interactions of the compounds; Kaempferol, Germacrene A and Elatine with selected bacterial drug targets are shown in (Table 5, Fig. 4 a-c). Highest docked score of − 9.2 kcal/mol was showed by elatine against Penicillin Binding Protein (PBP) and the lowest docked score of − 8.2 kcal/mol against the Elongation factor (EF-Tu). The docked structure was imaged to illustrate the ligand (Elatine) interactions with significant amino acids such as Tryptophan (TRP-374), Glutamate (GLU-378), Tyrosine (TYR-568), Threonine (THR-566) and Leucine (LEU-565) of Penicillin Binding Protein (PBP) through Vander Waal forces as well as hydrogen bonding. Ligand (Kaempferol) interacts with significant amino acids such as Phenylalanine (PHE-450), Isoleucine (ILE-371), Asparagine (ASN-377), Serine (SER-337), Lysine (LYS-340) and Arginine (ARG-372), Glutamine (GLN-447) of Penicillin Binding Protein (PBP). The best pose for each molecule was considered to investigate the intramolecular correlations. The docking of ligand elatine with Elongation Factor EF-Tu indicated the binding interactions with significant and functionally relevant amino acids such as Arginine (ARG-204), Alanine (ALA-205), Glutamate (GLU-203), Asparagine (ASN-13), Glycine (GLY-371), Glutamine (GLN-97) and Lysine (LYS-208). The docking studies of kaempferol ligand with elongation factor EF-Tu indicated the binding interactions with significant and functionally relevant amino acids such as Aspartate (ASP-99), Histidine (HIS-11), Glutamate (GLU-201), Asparagine (ASP-13), Proline (PRO-202), Arginine (ARG-204) and Lysine (LYS-208). The docked interaction studies of kaempferol with the dihydropteroate synthase (DHPS) displayed additional pi-cation/anion/alkyl binding, which established interaction with primary amino acids such as Asparagine (ASP-269), Alanine (ALA-270), Lysine (LYS-19) and Serine (SER-262), Histidine (HIS-58) and Glutamate (GLU-18). Furthermore, three targeted fungal proteins were also docked with Elatine, Kaempferol and Germacrene A, and binding affinity results are shown in Table 5. Elatine had showed the highest binding affinity against beta-tubulin with a docked score of − 8.8 kcal/mol followed by ABC transporters (− 6.9 kcal/mol) and 1,3-beta glycan (− 6.7 kcal/mol). The number of hydrogen bonds and amino acid residues involved in the best compound's ligand–protein interaction with three distinct fungal targets is given in (Fig. 4a–c). The docking of Elatine with ABC transporters had showed interaction with amino acids like Leucine (LEU-42), Glycine (GLY-39) and Isoleucine (ILE-40). The docking of kaempferol with ABC transporters had shown interaction with amino acids like Lysine (LLY-188), Leucine (LEU-184), Valine (VAL-49), Phenylalanine (PHE-196) and Isoleucine (ILE-206). Docked interactions of ligand (Elatine) with Beta-Tubulin had shown the interactions with amino acids such as Serine (SER-160), Leucine (LEU-163), Threonine (THR-166), Tyrosine (TYR-167), Proline (PRO-169) and Alanine (ALA-236). Docked interactions of ligand (Kaempferol) with beta- tubulin had shown the interactions with amino acids such as Valine (VAL-79), Tryptophan (TRP-96), Luecine (LEU-87), Alanine (ALA-93 and Isoleucine (113). Elatine interacts with target protein beta-glycan through various amino acids such as Serine (SER-160), Arginine (ARG-175), Tyrosine (TYR-167), Threonine (THR-167), Leucine (LEU-237) and Proline (PRO-169). Kaempferol interacts with target protein beta- glycan through various amino acids such as Lysine (LYS-313), Glutamate (GLU-259), Tyrosine (TYR-159) and Phenylalanine (PHE-305). Previous reports also revealed excellent interaction of naturally isolated compounds from endophytic *Penicillium setosum* against various microbial drug target proteins^42^. The details regarding the number of hydrogen bonds shared with the amino acid/nucleotide residues at the active site regions of target proteins are represented in Table 6.

**Table 5:** The binding affinity of selected compounds against microbial proteins.

Ligands	Dihydropteroate synthase (kcal/mol)	Penicillin binding protein (kcal/mol)	Elongation factor EF-Tu (kcal/mol)
Kaempferol	− 8.0	− 7.9	− 6.1
Germacrene A	− 7.2	− 7.1	− 6.2
Elatine	− 8.5	− 9.2	− 8.2

Antifungal proteins			
	1,3 β-glycan (kcal/mol)	ABC transporter (kcal/mol)	Beta-tubulin (kcal/mol)
Kaempferol	− 6.7	− 7.3	− 7.1
Germacrene A	− 7.7	− 8.7	− 8.1
Elatine	− 6.7	− 6.9	− 8.8

**Table 6:** Lists the interacting amino acid residues involved in the ligand–protein interaction of the selected compounds against six different targets and bond lengths between the amino acid of the target protein and ligand.

Ligand	Target proteins	Bond lengths	Interacting amino acids
Kaempferol	Penicillin-binding protein (PBP)	3.40, 2.02, 2.70, 1.94, 1.88, 2.53	ILE-371, PHE-450, ASN-377, SER-337, LYS-340, ARG-372 and GLN-447
Kaempferol	Elongation factor Tu (ETU)	5.29, 2.29, 2.05, 2.89, 2.90, 2.14	ASP-99, HIS-11, GLU-201, ASP-13, PRO-202, ARG-204 and LYS-208
Kaempferol	Dihydropteroate synthase (DHPS)	2.40, 2.13, 3.74, 4.10, 5.32, 4.55, 4.58	ASP-269, ALA-270, LYS-19, SER-262, HIS-58 and GLU-18
Kaempferol	ABC transporter	5.47, 2.23, 5.05, 4.90, 5.48	LEU-184, ILE-206, VAL-49, PHE-196 and LYS-188
Kaempferol	1,3 β-glycan	2.58, 4.44, 5.28, 3.56, 4.66, 4.69, 5.50	ASN-199, GLU-259, LYS-313, PHE-305, ASN-159 and ILE-200
Kaempferol	Beta-tubulin	3.64, 3.46, 2.17, 2.56, 4.44	VAL-79, TRP-96, LEU-87 and ALA-93
Germacrene A	Penicillin-binding protein (PBP)	4.35, 4.75, 2.05	LEU-302 and LYS-615
Germacrene A	Dihydropteroate synthase (DHPS)	4.77, 5.65, 4,19, 3.01	MET-200, ARG-201 and LEU-197
Germacrene A	Elongation factor Tu (ETU)	3.70, 4.86, 0.58, 3.91	PHE-218, ILE-220, VAL-226 and THR-228
Germacrene A	ABC transporter	2.04, 5.02, 4.54, 5.16, 5.84	LEU-184, ILE-194, VAL-49, PHE-181 and GLN-185
Germacrene A	1,3 β-glycan,	1.45, 2.35, 5.06, 4.33, 5.01	PHE-205, ARG-123, ASP-208, GLU-118, TYR-159 and TYR-201
Germacrene A	Beta-tubulin	2.14, 5.30, 5.39, 1.44, 5.20, 1.91, 3.88	VAL-79, LEU-87, TRP-96, ALA-93 and ILE-113
Elatine	Penicillin-binding protein (PBP)	1.80, 1.57, 2.05, 2.88, 3.58	TRP-374, GLU-378, TYR-568, THR-566 and LEU-565
Elatine	Elongation factor Tu (ETU)	2.43, 2.79, 2.59, 3.46, 2.65, 1.95	GLU-203, ASN-13, ARG-204, ALA-205, LYS-208, GLN-97 and GLY-371
Elatine	Dihydropteroate synthase (DHPS)	2.78, 3.33, 5.09, 3.17	TYR-103, ASN-147, ILE-122, PHE-123, TRP-189, ILE-150, ARG-148 and ALA-190
Elatine	ABC transporter	3.32, 4.66, 2.39, 2.90, 2.14, 3.87	LEU-42, ILE-219, ASN-41 and GLY-39
Elatine	1,3 β-glycan	2.02, 2.04, 3.25, 3.30, 3.87, 4.99	LEU-237, ARG-175, SER-160, THR-166, TYR-167, PRO-169 and ALA-236
Elatine	Beta-tubulin	3.54, 3.79, 3.38, 3.26, 5.35, 5.36	SER-160, LEU-163, TYR-167, THR-166, PRO-169 and ALA-236

### Molecular dynamic simulation

**Figure 5:**
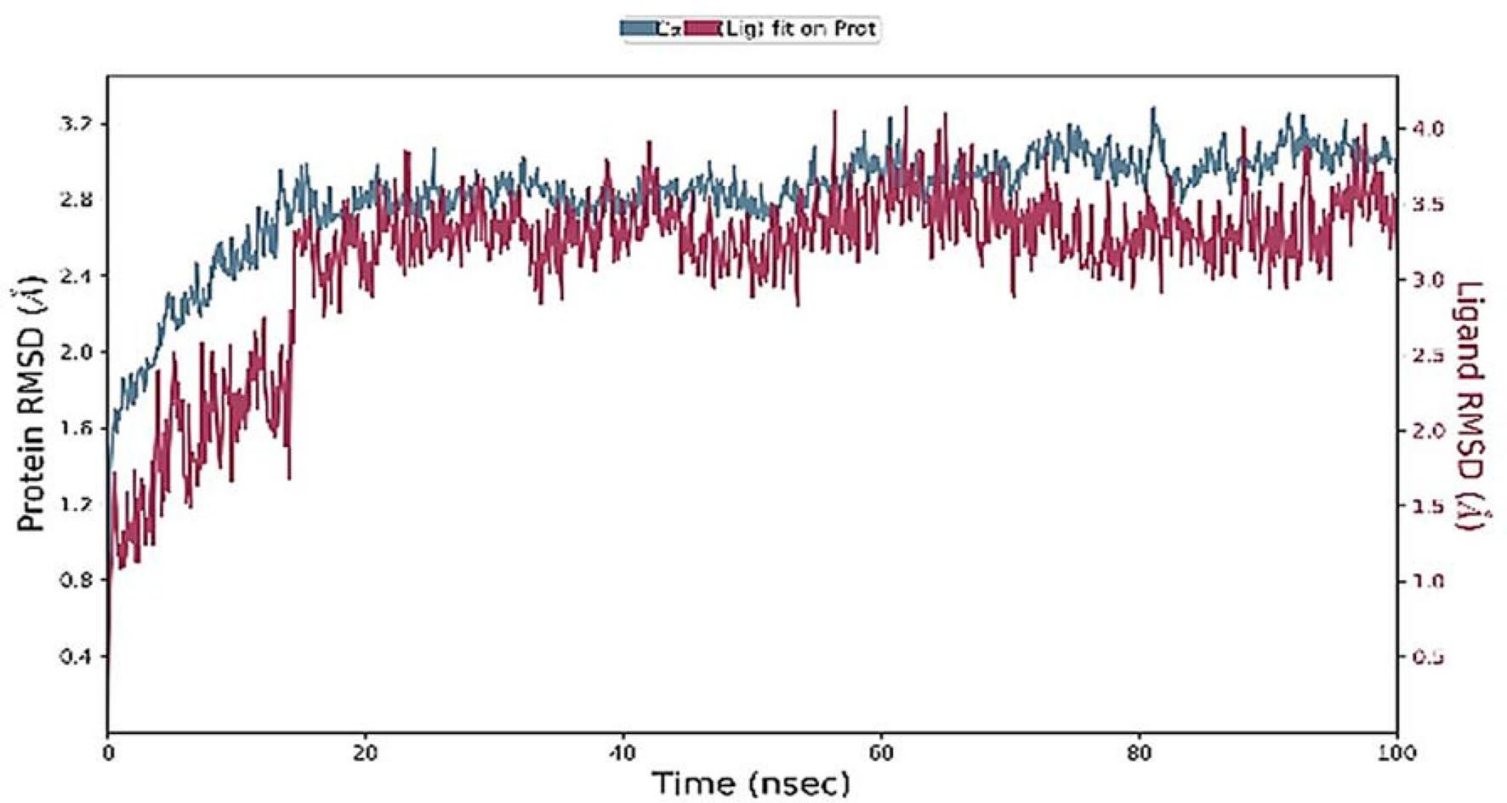
Protein–ligand RMSD plot.

**Figure 6:**
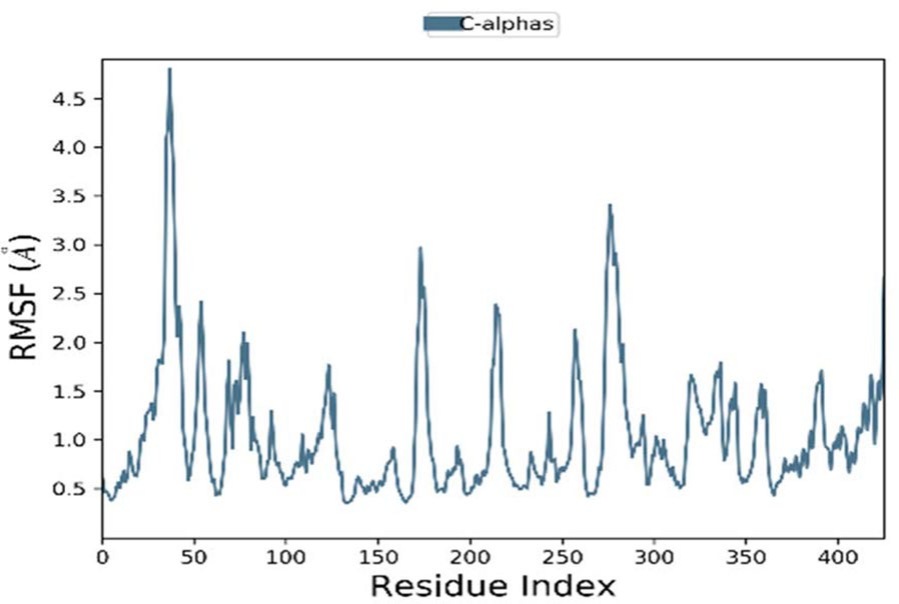
Protein–RMSF plot.

The docking interpretation was validated using the dynamic simulation Desmond Schrodinger tool. MD simulation was carried out to investigate the stability of elatine in the active pocket of beta-tubulin. MD simulation determines the stability and convergence between ligand and target protein. Two independent MD simulation of 100 ns each was run. The root mean square deviation (RMSD), root mean square fluctuations (RMSF), radius of gyration (Rg) and hydrogen bond distances were analysed to establish the related stability (Supplementary file). The RMSD provides information on the stability of the complex. The RMSD of the ligand obtained after least square fit shows few fluctuations during the first 10 ns, and then remained stable until 100 ns time Fig. 5. The internal motion and fluctuations of the residues were analysed by calculating the RMSF. Higher fluctuations were observed to residues forming the loop 1 (Fig. 6) at the region 45 to 50. Other region observed to have large fluctuations is 270 to 290.

**Figure 7:**
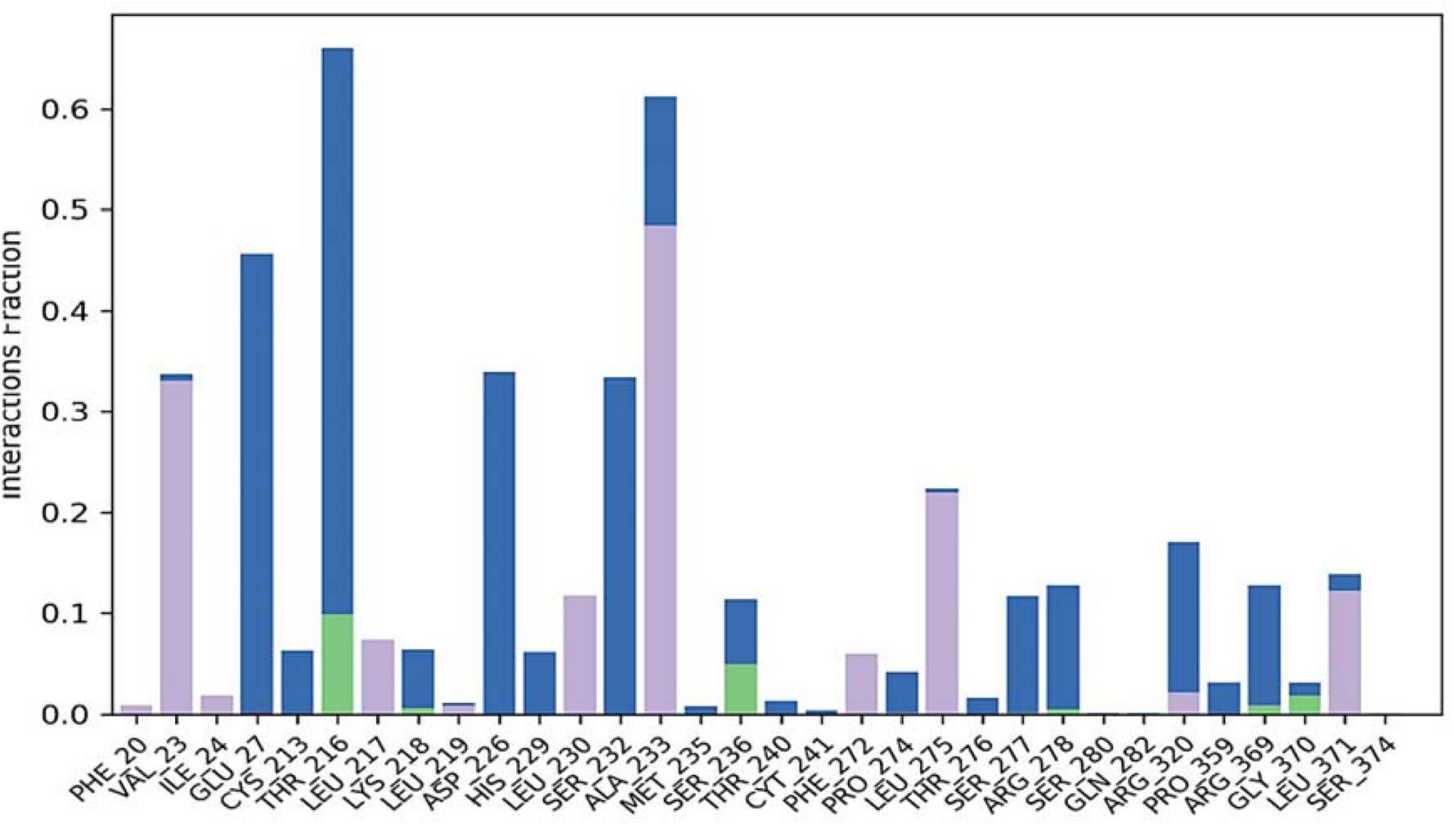
Hydrogen bond contact analysis of lead compound and elatine–protein complexes. Various intermolecular interactions made by elatine–protein amino acid residues with lead ligand during molecular dynamics simulations. Bar colors: Hydrogen bond (Green), Hydrophobic (Purple), Water bridge (Blue).

The ligand exposed high water bridges, hydrogen bonding and hydrophobic interactions with the amino acid residue of Threonine 216, Alanine 233, Glutamate 27 as shown in Fig. 7. The protein–ligand contact of amino acid residues of ligand–protein made hydrogen bond contacts with the ligands throughout the simulation time. The overall results of the molecular dynamics showed that elatine compound was stable and interacted with the protein during the simulation period. These results were very well correlated with the results of the molecular docking.

## Discussion

Phytochemical analysis of *G. wallichainum* extracts had shown presence of various secondary metabolites such as alkaloid, phenolic, flavonoids, glycosides, saponins and tannins, as depicted in Table 2. Main phytochemicals such as alkaloids have antimicrobial activity and analgesic; tannins and flavonoids contribute as antibacterial and antioxidant agents^43^; saponins have anticancer, antibacterial, anti-diabetic and anti-inflammatory activities^44^. The abundance of these phytochemicals in the *G. wallichianum* extracts might contribute to its therapeutic potential. Several research studies have revealed the antimicrobial activities of the genus *Geranium*^45–47^. In this respect, it was essential to investigate the antimicrobial potential of the *G. wallichianum*, a critical medicinal species of the genus *Geranium*. The antimicrobial activity may be caused by bioactive compounds, namely phenolics, flavonoids and alkaloids compounds^48^. Tentative identification of the various extracts by LC–MS investigated many compounds with antimicrobial potential, namely kaempferol^49^, quercetin^50^, germacrene D^51^, caffeic acid^52,53^ and p-coumaric acid^54^. The minimum inhibitory concentration (MIC) performed the preliminary screening of antimicrobial activity. Based on the obtained results (Table 4), the highest antimicrobial activity was observed in the case of ethyl acetate extract against various bacterial strains compared to methanol, ethanol and petroleum ether. Plant extracts had showed less sensitivity against three fungal strains. In general, extracts of ethyl acetate had shown the most promising antibacterial potential against *M. luteus* with MIC value of 3.5 μg/ml followed by *H. influenzae, S. pneumoniae, K. pneumoniae, N. mucosa* and *E. coli* (MIC values*:* 6.25, 12.5, 25, 25, and 100 μg/mL respectively), whereas *Candida* species were less sensitive to ethyl extract with MIC values of 400 μg/ml respectively. Results of methanol and ethanol extracts were moderately effective against the different microbial strains. Petroleum ether extracts had showed maximum antimicrobial activity against the *M. luteus* with MIC value of 1.56 μg/ml and the least antimicrobial potential against the *E. coli* with an MIC value of 100 μg/ml. However, all the four plants extract of *G. wallichianum* had showed significant antimicrobial activity against the selected microbial pathogens. The antimicrobial potential of various extracts of *G. wallichianum* might be attributed to the presence of phytocompounds; flavonoids, phenolic acids, alkaloids and diterpenoids. Hence, the results of antimicrobial activity obtained in the present study of four *G. wallichianum* extracts were correlated with their total polyphenolic contents. Besides, the research conducted confirmed that phenolics were the most significant active compounds against bacterial infection. Previous reports on antimicrobial activities of various species of *Geranium* genus had also revealed excellent results on various bacterial and fungal strains. In silico investigations have been effectively used to predict the theoretical ligand and target interactions for more complete understanding of the molecular basis of natural product biological activity.

**Figure 8:**
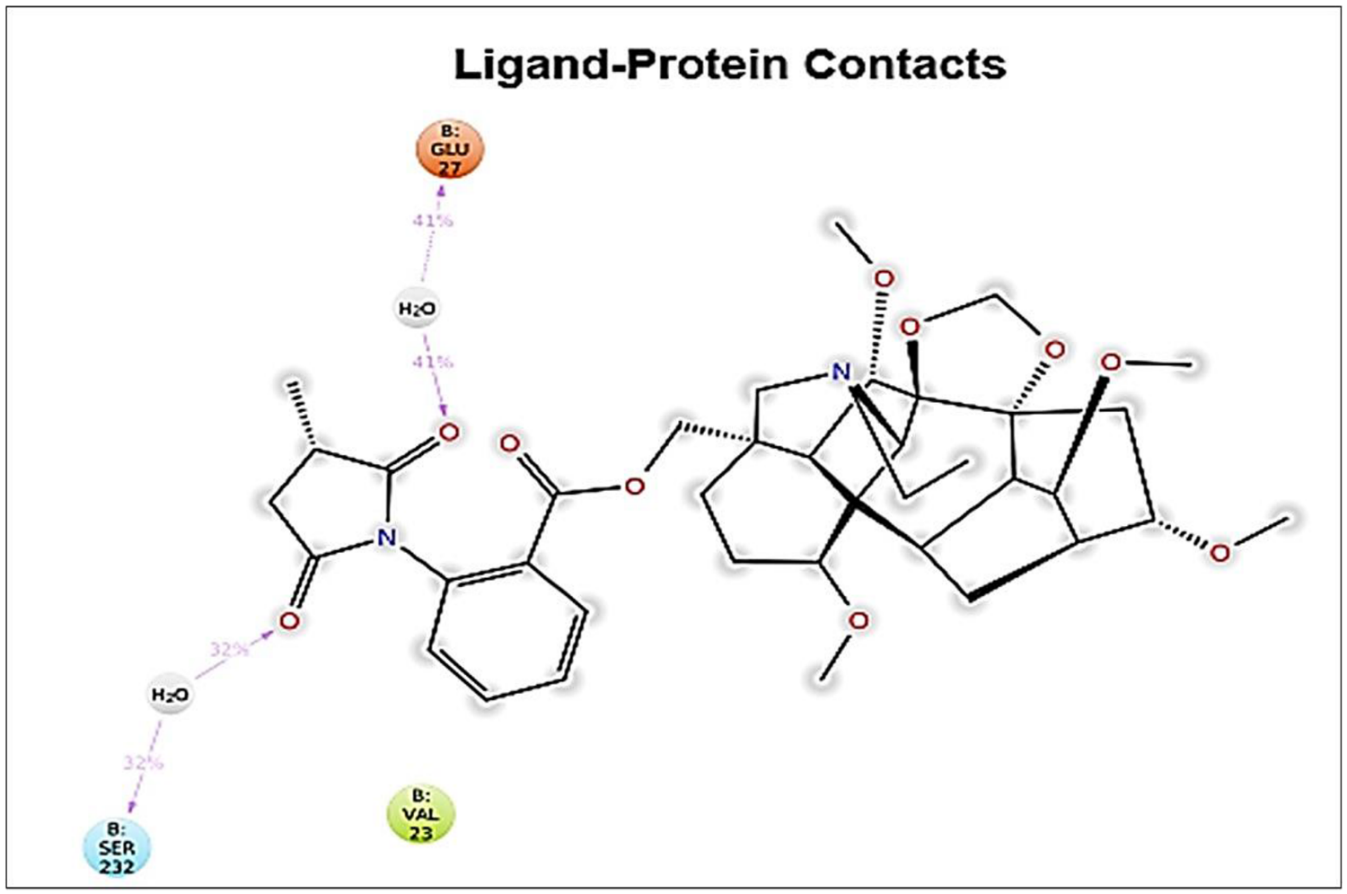
A schematic of detailed ligand atom interactions with the protein residues. Interactions that occur more than **30.0%** of the simulation time in the selected trajectory (0.00 through 100.00 nsec), are shown.

Based on the virtual screening, three compounds viz. Kaempferol, Germacrene A and Elatine were obtained through LC–MS analysis. Ligands identified through LC–MS from *G. wallichainum* were docked to the active sites of various microbial drug target proteins using AutoDock4.2^55^. Further detailed investigation on interaction of the obtained compounds against different targets involved in various biochemical processes of microbial growth were evaluated using the *in-silico* approach. Binding energy is a function of the stability of the complex formed between ligand and target protein. It also optimizes new bonds that in turn may affect the biological activity of the resulting complex. To further display various interactions involved between ligands and target protein at the active site, the docked complexes were visualized through Discovery Studio Visualizer^56^. Among the three compounds, elatine showed highest binding affinity with pencillin binding protein (PBP) followed by kaempherol towards the selected drug targets of bacteria and fungi. There are many different targets through which an antibacterial compound can inhibit cell wall synthesis. Such mechanisms have been regarded as important antibacterial targets for years^57^. In bacterial cells, penicillin-binding proteins (PBPs) polymerize and modify peptidoglycan, the stress-bearing component of the bacterial cell wall. As part of this process, the PBPs help to create the morphology of the peptidoglycan exoskeleton together with cytoskeleton proteins that regulate septum formation and cell shape. Many natural compounds were reported to inhibit the synthesis of PBP^58,59^. Interestingly, elatine and kaempherol showed highest binding affinities as compared to already known natural inhibitors against PBP. The highest binding affinity of kaempherol was also supported by molecular simulation study on the interaction between tyrosinase and flavonoids from *Sea Buckthorn*^60,61^. Another well-known antimicrobial target is (DHPS) is an essential enzyme in the biosynthesis of dihydrofolate in microorganisms. DHPS is an important target for selective antimicrobial agents. Sulfonamides are the oldest synthetic, effective antimicrobial agents and they target DHPS in bacteria. Other inhibitors, such as diaminodiphenylsulfone (dapsone) or para-aminosalicylic acid (PAS), also inhibit DHPS and are effective against certain mycobacteria (*M. tuberculosis*, *M. leprae*). Both kaempherol (flavonoid) and elatine showed significant binding affinities with DHPS. Many known antibacterial drugs interfere with protein synthesis by binding with specific sites on ribosomes, which can also be considered as another important target^62^. Elongation factor Tu (EF-Tu) is responsible for attachment of aminoacylated tRNA to 16S rRNA A site of 30S rRNA, hence binding of antibacterial compounds to EF-Tu as well 16S rRNA A site leads to translational errors^63^. In the docking study, it was also found that elatine exhibits significant binding affinity with EF-Tu. The components identified from the antimicrobial active fraction are all reported as plant secondary metabolites. Many plants derived flavonoids have been reported for their broad-spectrum antibacterial action, but few reports are available on the identification of fungal derived antibacterial flavonoids together with detailed aspect of their mechanisms. The docking results obtained with the test ligands were compared with ciprofloxacin, a commercially available antibacterial drug. The docking of the ciprofloxacin against PBP showed a binding energy of − 8.04 kcal/mol. This interaction was achieved by van der Waals forces, pi-pi stacking, pi-alkyl, and alkyl interactions, which probably helped loperamide to intercalate at the binding site of PBP. But these are weaker interactions in comparison to the hydrogen bonds^64^. In fact, amongst all the intermolecular non-covalent interactions, hydrogen bonds play a central role in the binding of a ligand to the active site of the protein. In the MD simulation, the stable complex system was analyzed for the type of protein ligand interaction in 100 ns of simulation. The interaction with GLU-27, SER-232, and VAL-23 was the most frequent interaction that could be maintained during the simulation. GLU-27 was found to form hydrogen bonds with two hydroxyl groups ofElatine, 41% and 41%, over the 100 ns of simulation time. SER-232 was also found to interact by hydrogen bonding for 32% and 32%% through two hydroxyl groups of Elatine (Fig. 8). These could be considered key residues for the interactions. Conclusively, the in silico molecular docking results describe the interaction of Kaempferol, Germacrene A and Elatine with the penicillin binding protein (PBP), dihydropteroate synthase (DHPS), elongation factor-Tu (Eu-Tu), 1,3 β-glycan, ABC transporter, and beta-tubulin confirm our finding of the plant extract possess antimicrobial activity.

## Conclusion

The present study reflects that *G. wallichianum* has significant antimicrobial activity against various microbial strains. Molecular binding interaction of *in-silico* data demonstrated that elatine, kaempferol, and germacrene A have more specificity towards the penicillin binding protein (PBP) and beta tubulin binding sites. They could be compounds with a potent antimicrobial activity. This can be further exploited to provide insights into the mechanism of action of potential antimicrobial drugs for resistant bacterial and fungal strains.

## Supplementary Information

Supplementary Information.

Supplementary Legends.

## Data Availability

All data generated or analysed during this study are included in this published article [and its supplementary information files].
