# Supplementary material for: Molecular docking analysis and evaluation of the antimicrobial properties of the constituents of Geranium wallichianum D. Don ex Sweet from Kashmir Himalaya
Source: Sci Rep. 2022 Jul 22;12:12547. doi: 10.1038/s41598-022-16102-9 (PMC9307801; doi:10.1038/s41598-022-16102-9)
Supplement: Supplementary file 1 — Supplementary Information. [file 41598_2022_16102_MOESM1_ESM.pdf]

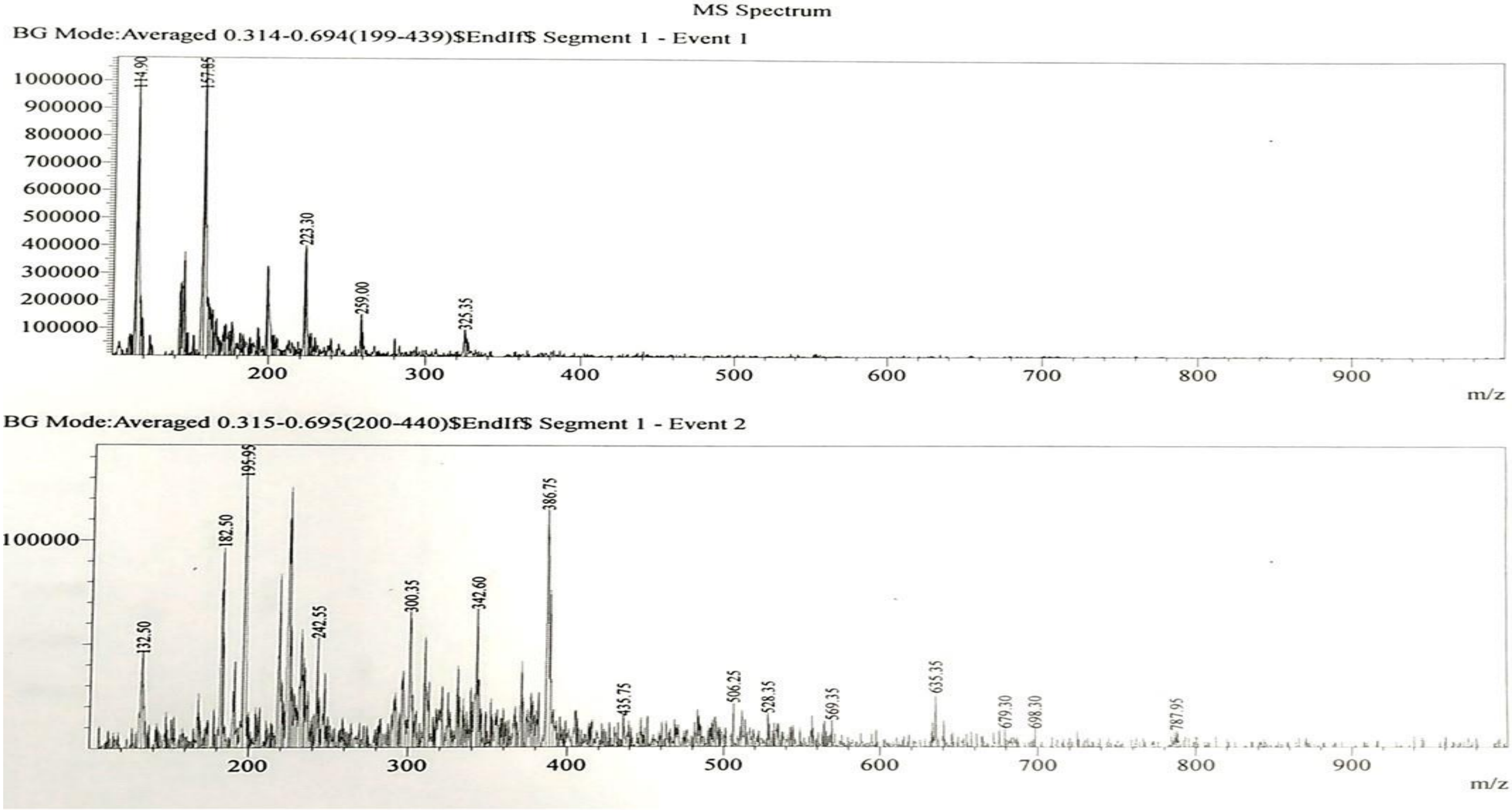

Fig. S1. LC-MS-ESI-MS chromatograms of reference compounds using Nexera in Ethanolic extract

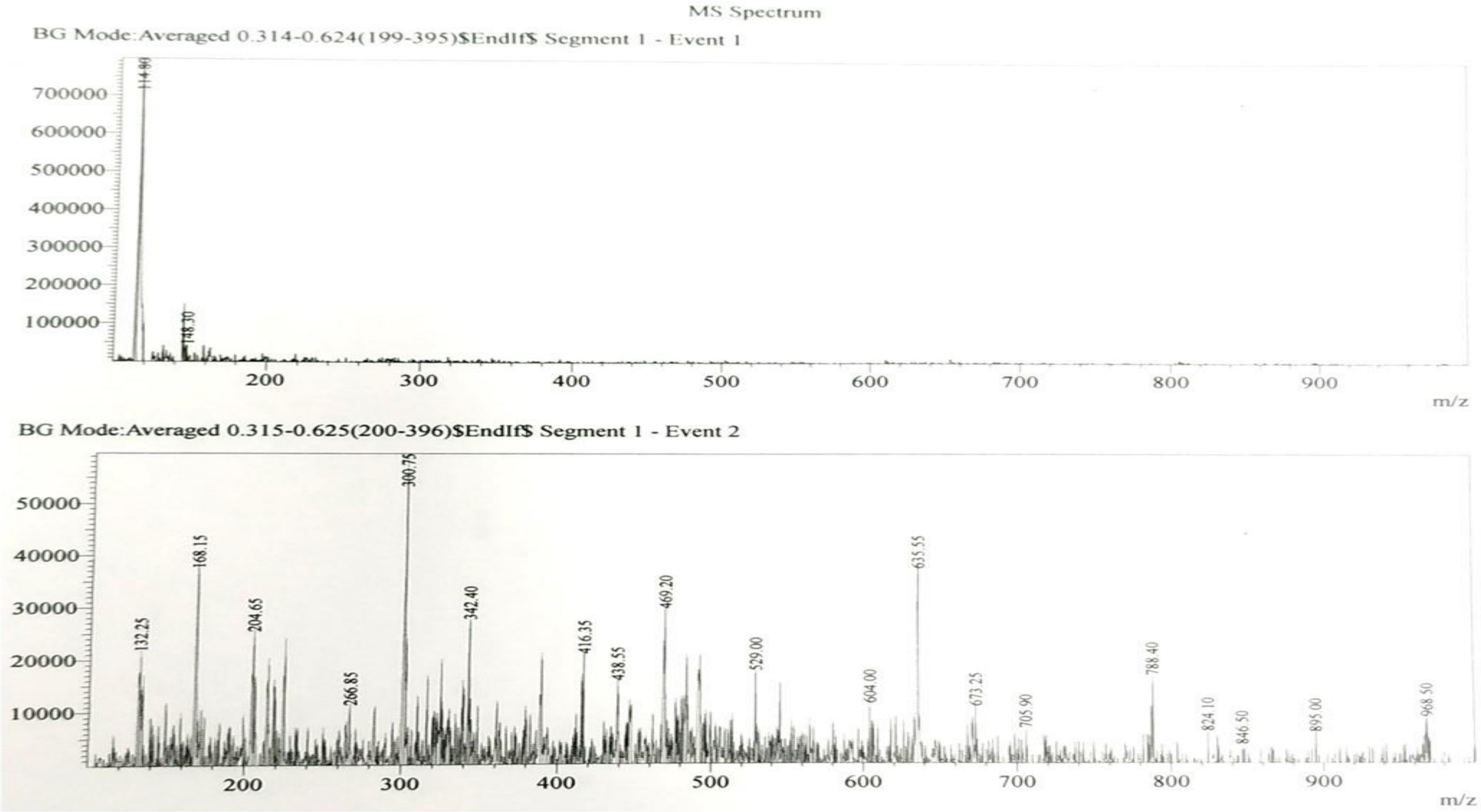

Fig. S2. LC-MS-ESI-MS chromatograms of reference compounds using Nexera in Ethyl acetate extract

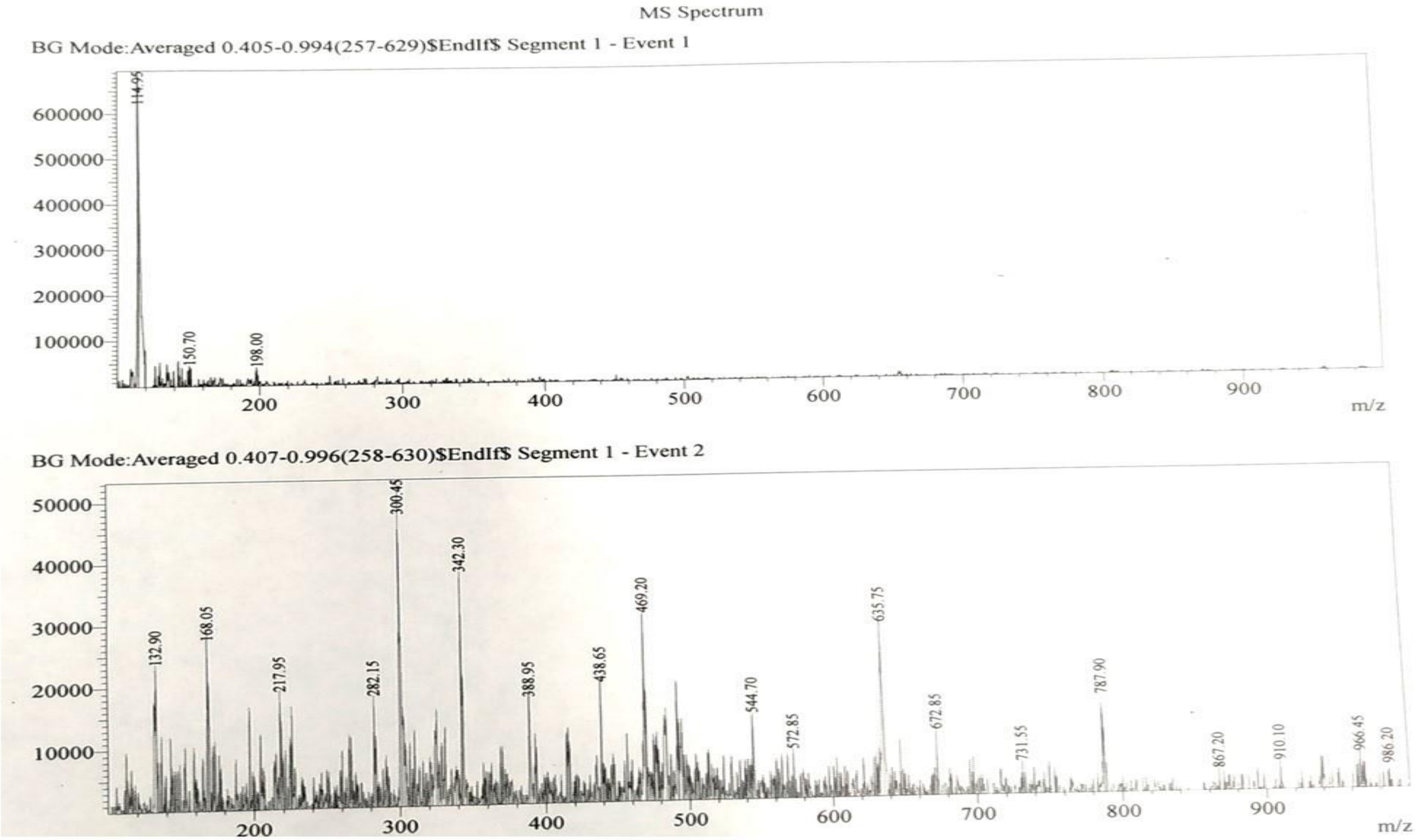

Fig. S3. LC-MS-ESI-MS chromatograms of reference compounds using Nexera in Petroleum ether extract

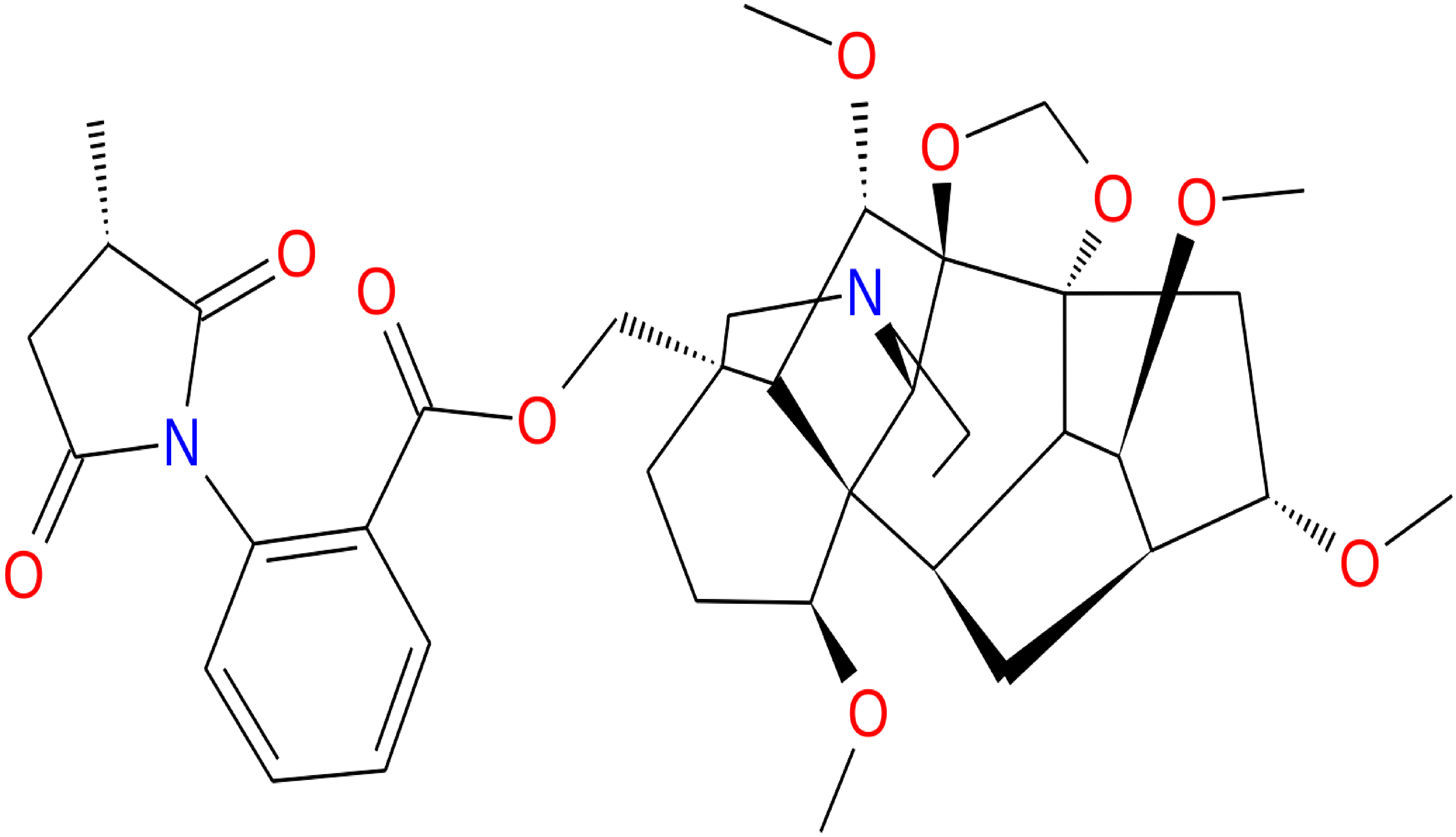

Fig. S4. 2d structure of Ligand (Elatine)

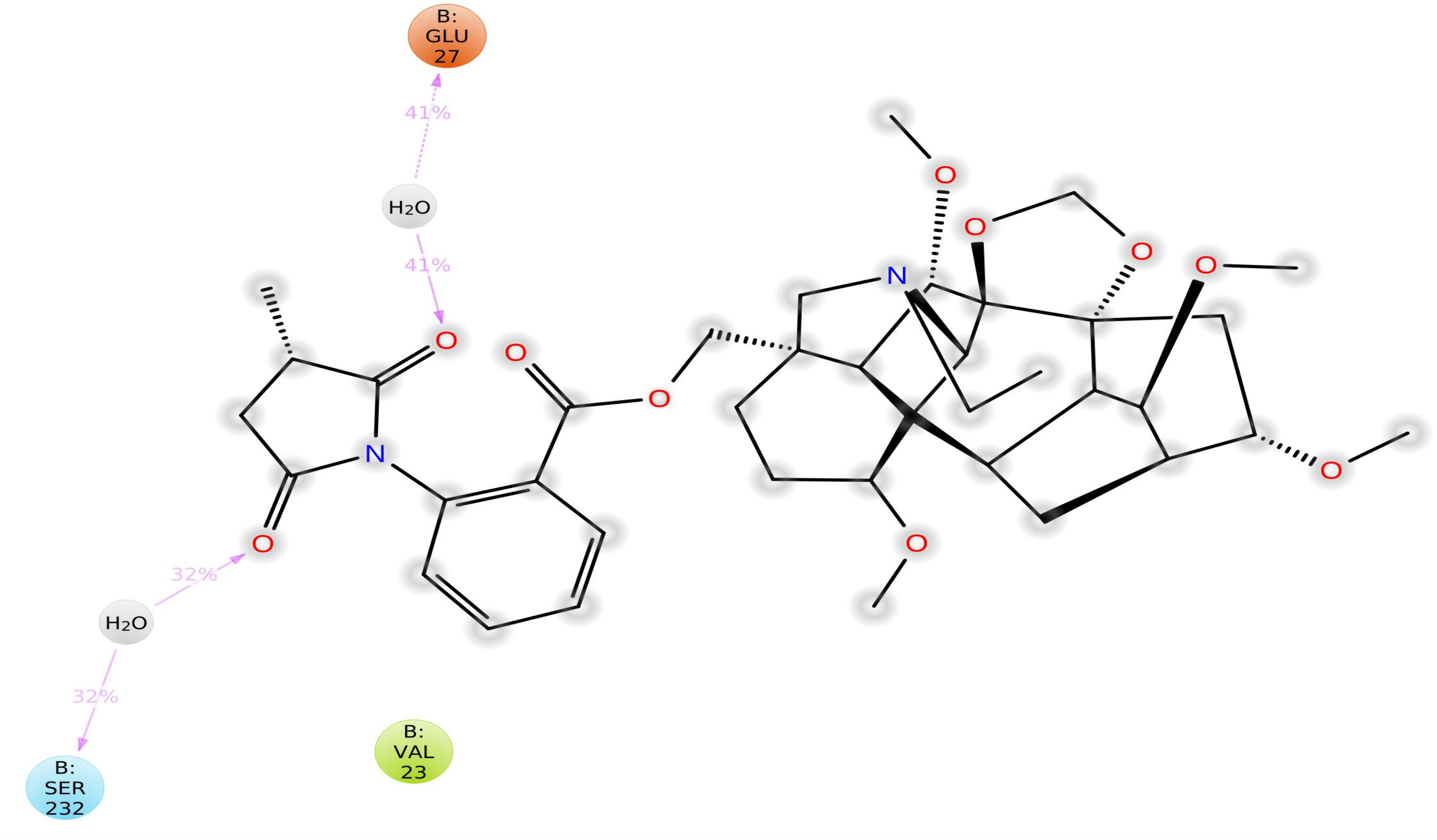

Fig. S5. Ligand (Elatine)-Protein (Beta-Tubulin) Contacts

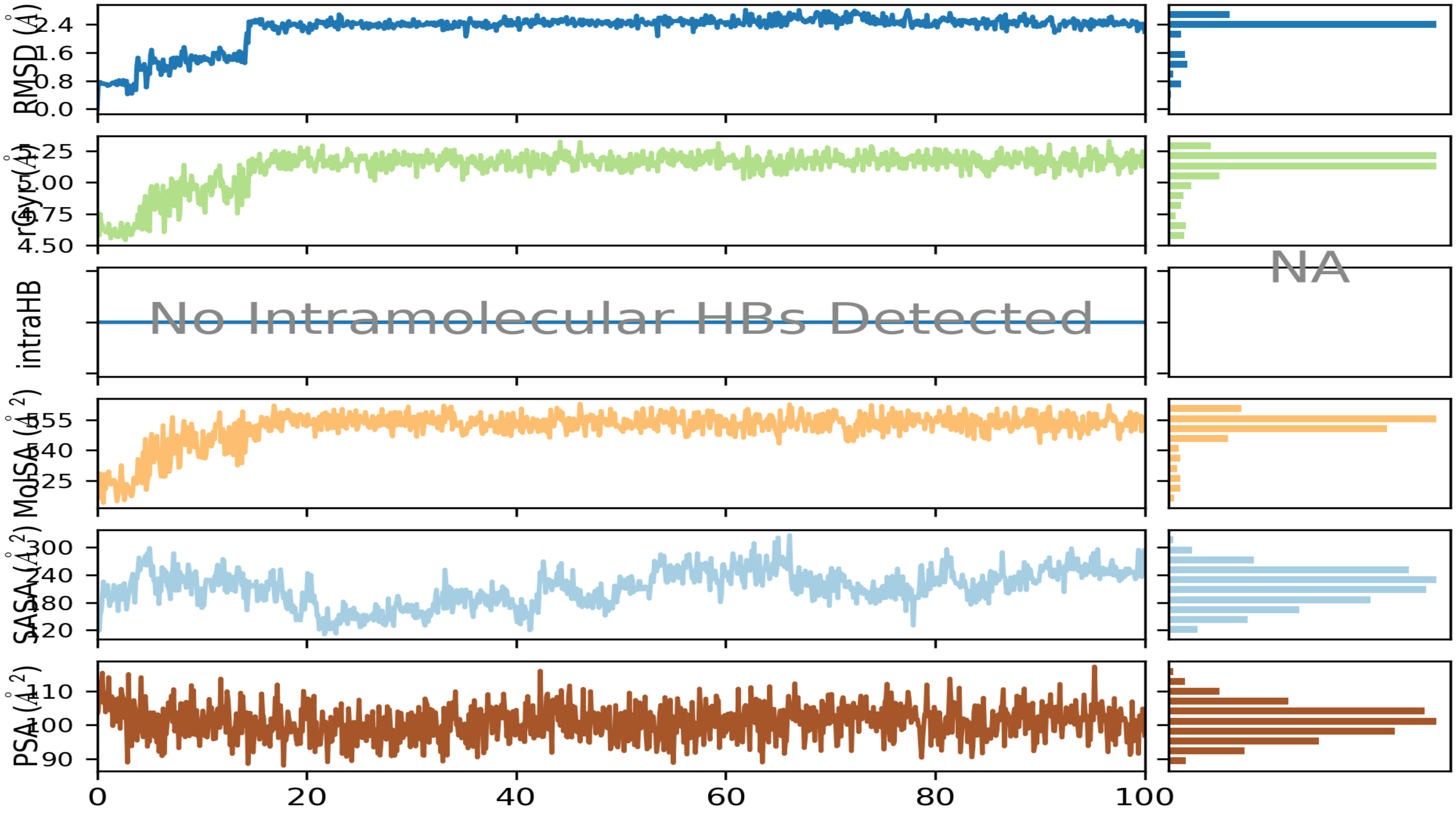

Fig. S6. Root Mean Square Deviation, Intramolecular Hydrogen Bonds, Radius of Gyration, Solvent Accessible Surface Area, Molecular surface Area, and Polar Surface plots of the hit compound (Elatine).

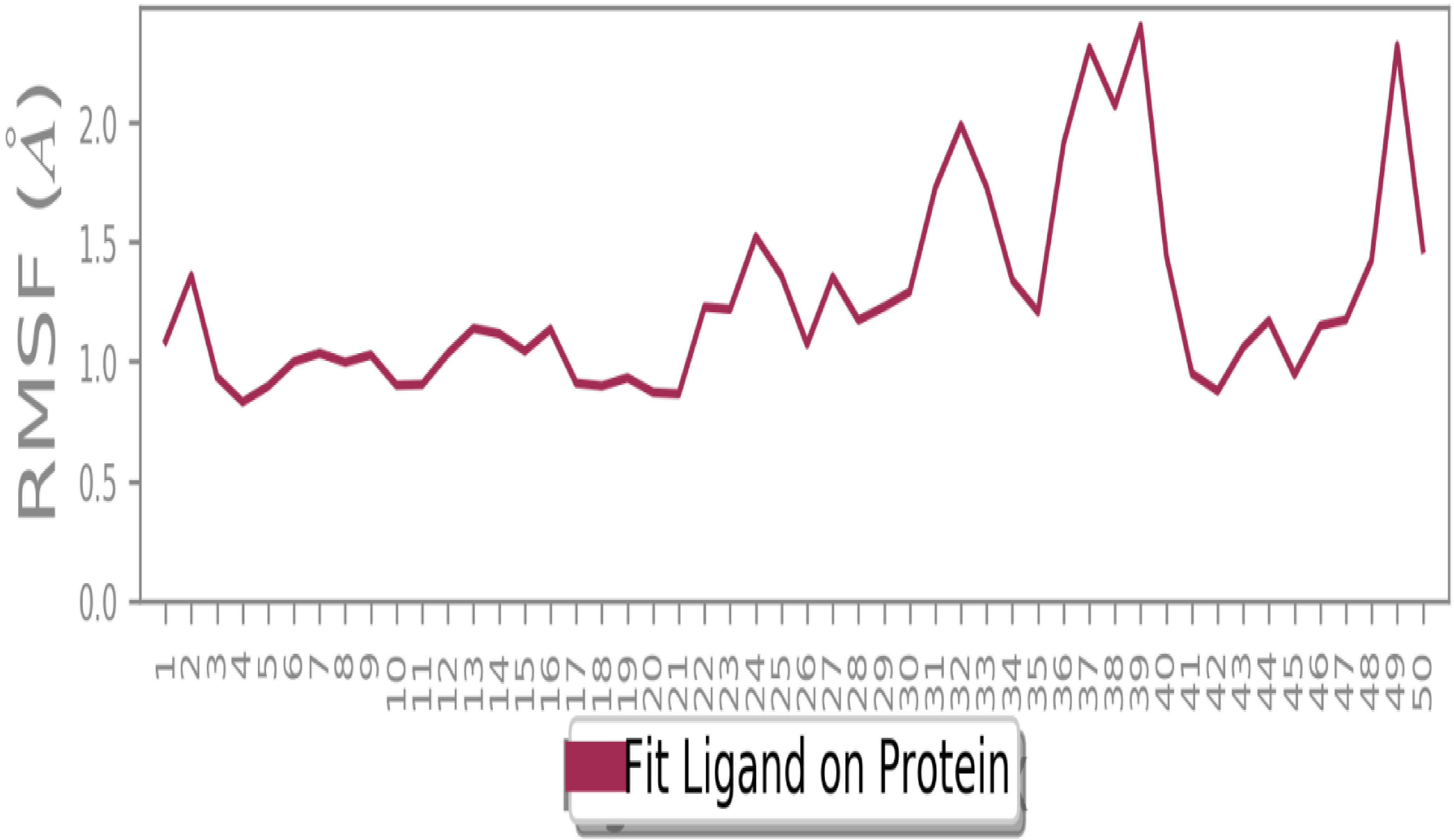

Fig. S7. The Ligand Root Mean Square Fluctuation (L-RMSF)

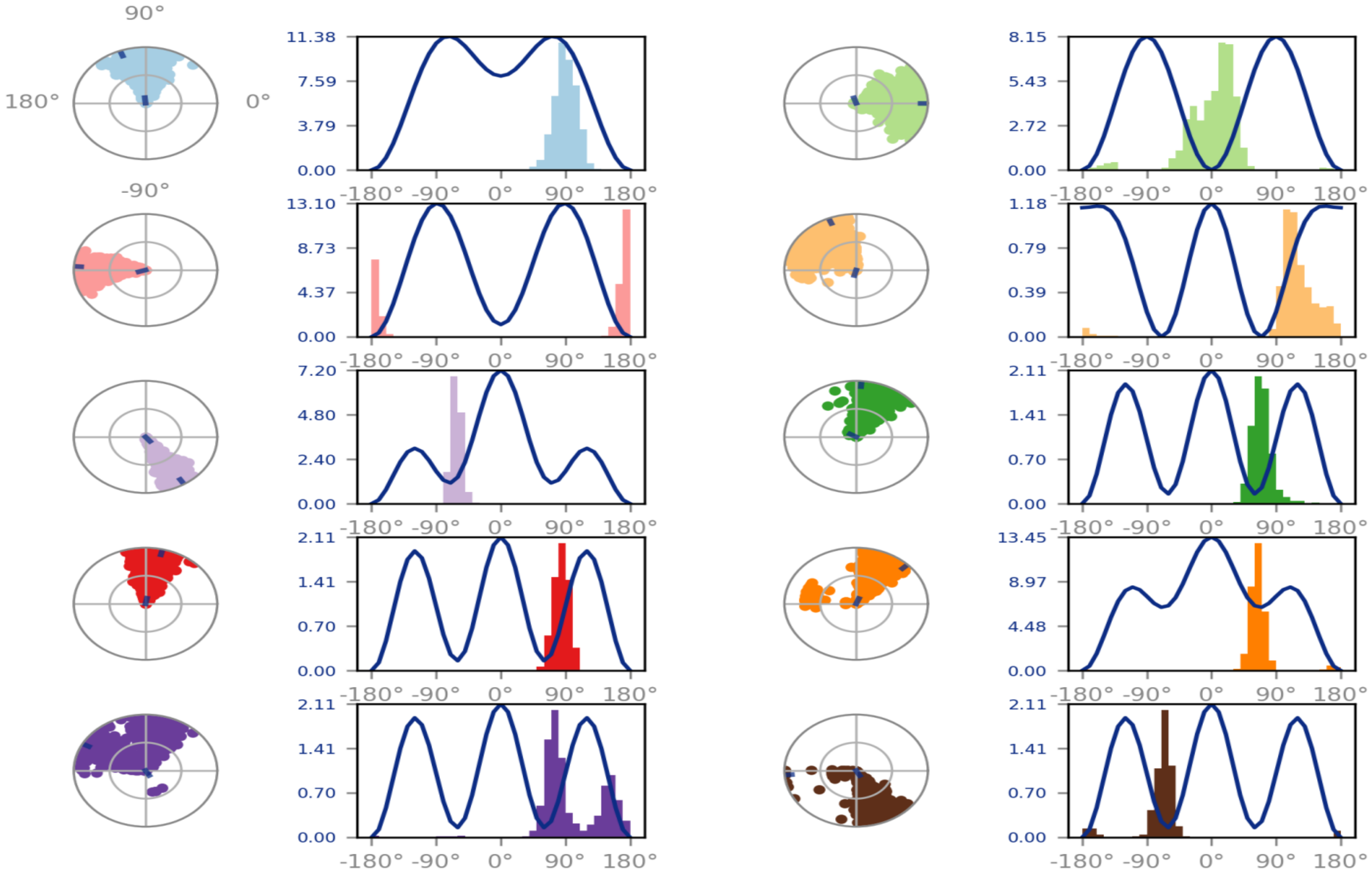

Fig. S8. Rotatable bonds (RBS) in the ligand throughout the simulation trajectory (0.00 through 100.00 nsec).

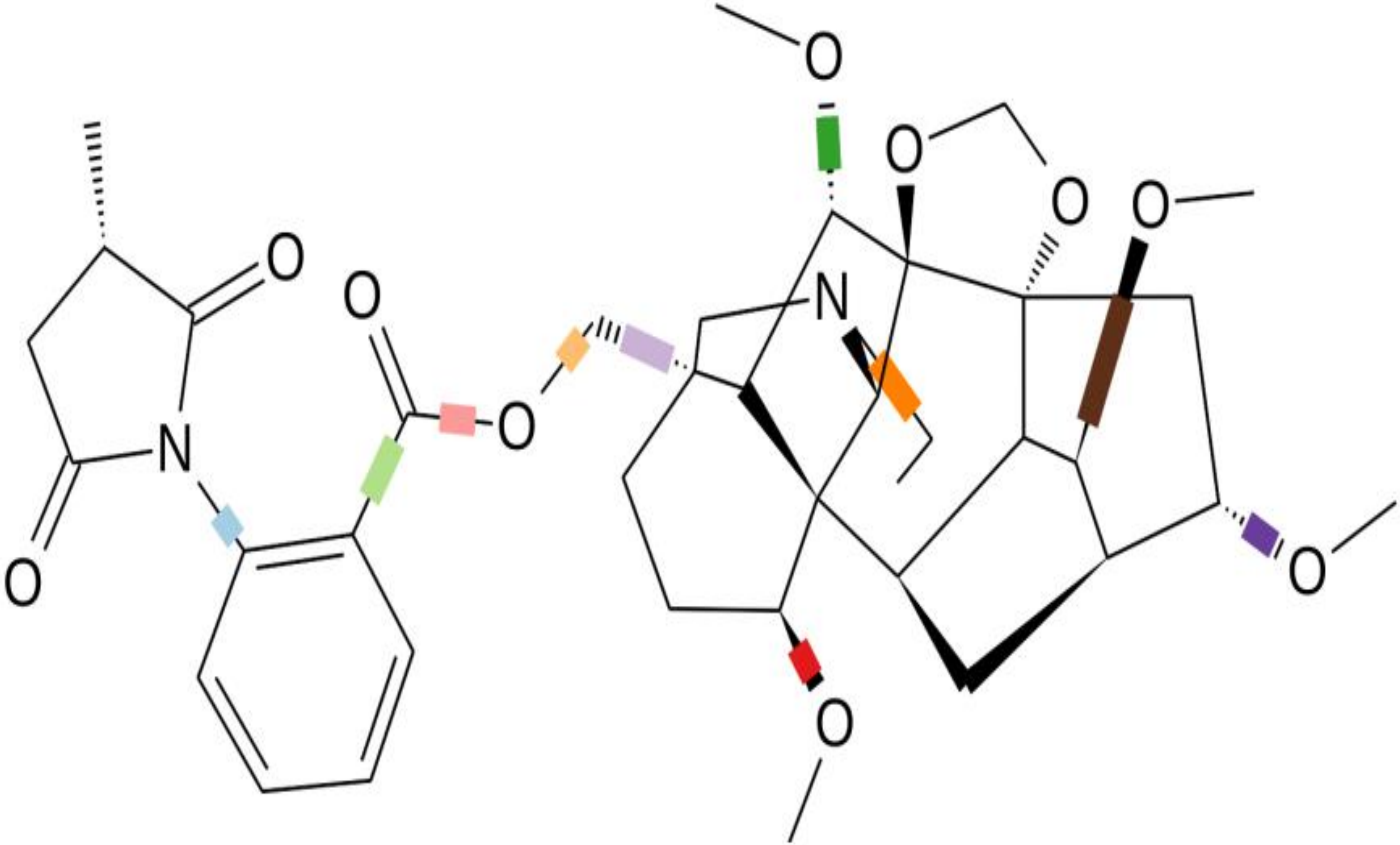

Fig. S9. Ligand Torsion Profile

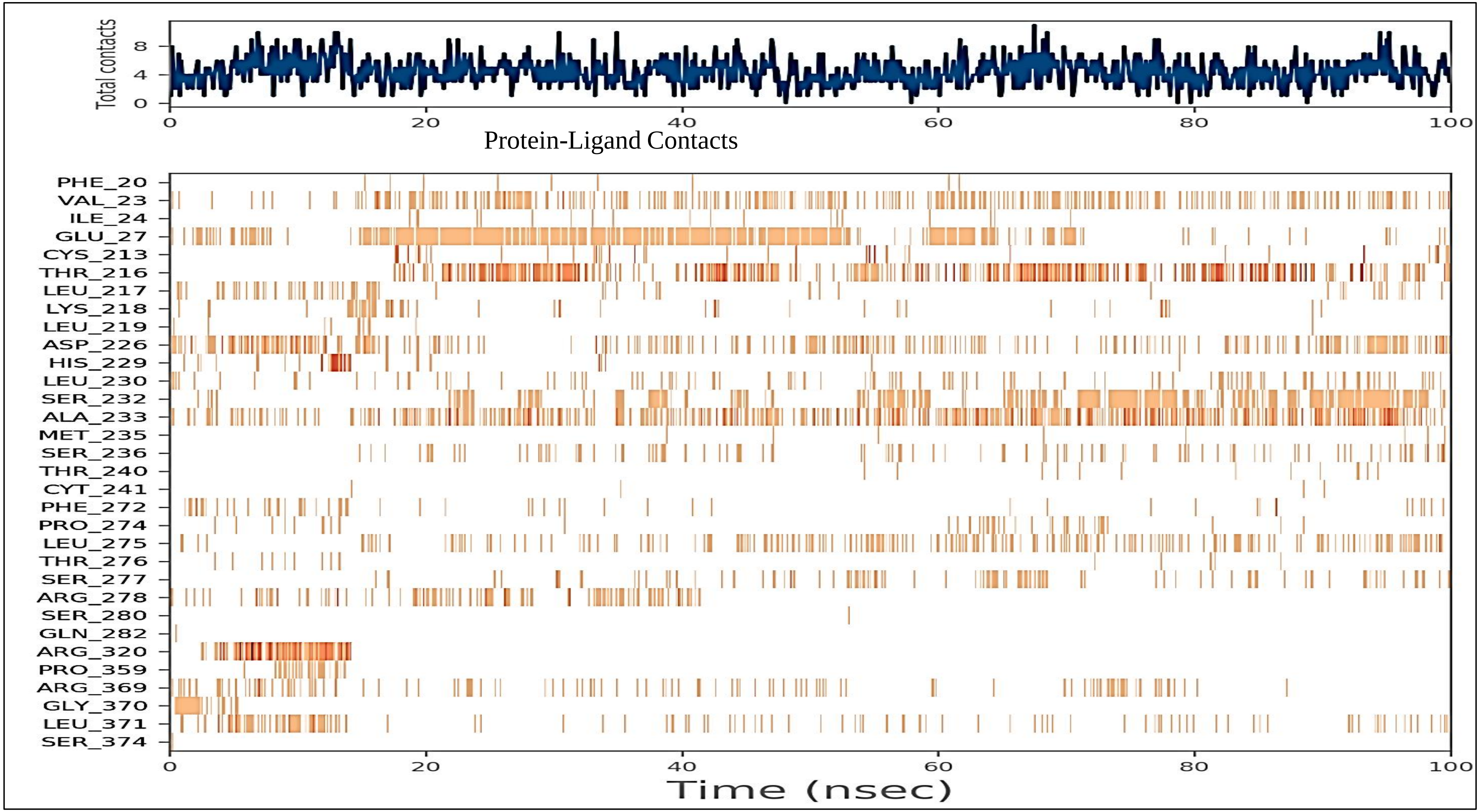

Fig. S10. The top panel shows the total number of specific contacts the protein makes with the ligand over the course of the trajectory. The bottom panel shows which residues interact with the ligand in each trajectory frame. Some residues make more than one specific contact with the ligand, which is represented by a darker shade of orange, according to the scale to the right of the plot.

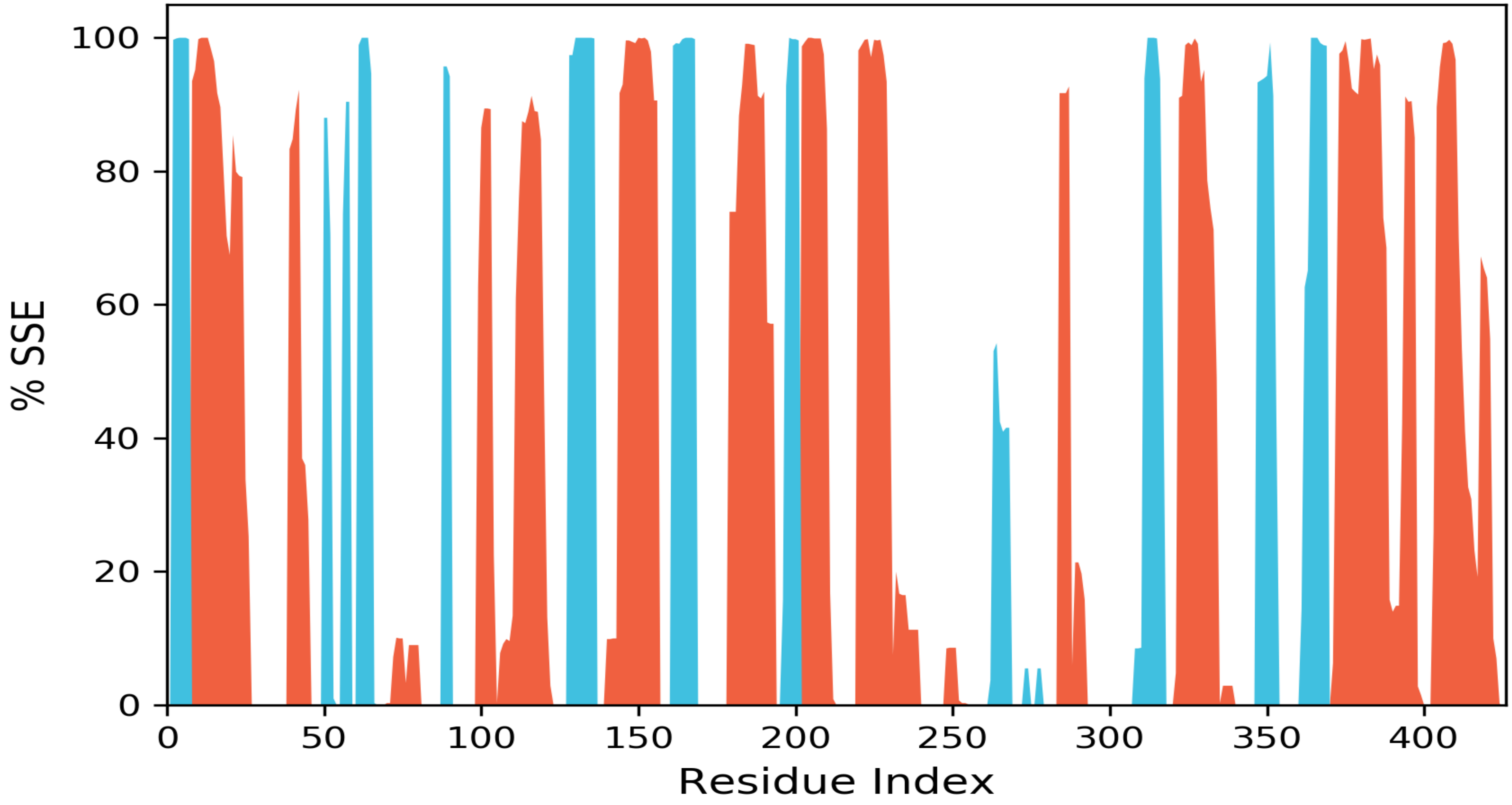

Fig. S11. **Protein Secondary Structure**

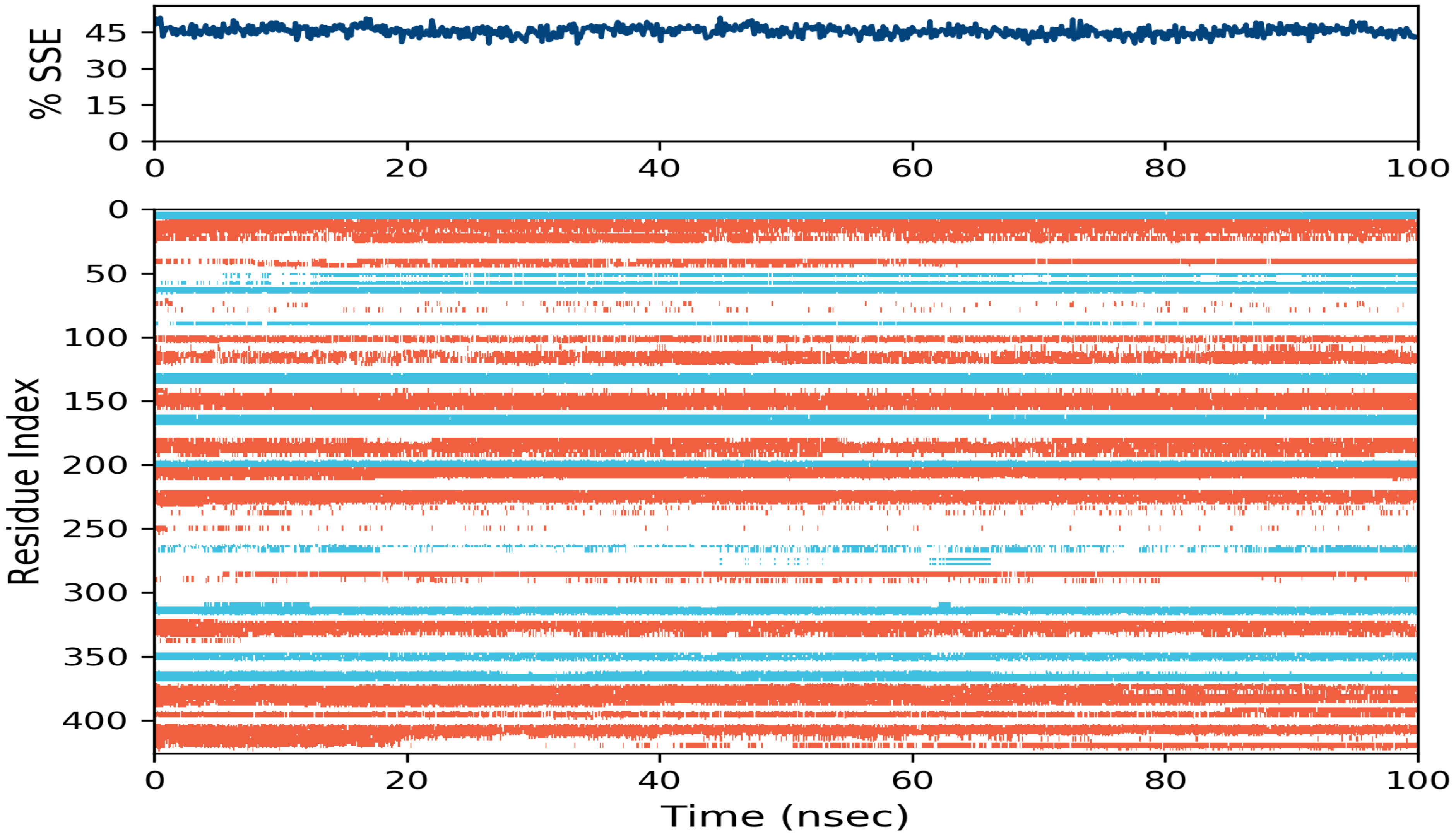

Fig. S12. Protein secondary structure elements (SSE) like alpha-helices and beta-strands are monitored throughout the simulation. The plot above reports SSE distribution by residue index throughout the protein structure. The plot below summarizes the SSE composition for each trajectory frame over the course of the simulation, and the plot at the bottom monitors each residue and its SSE assignment over time.

Supplementary data: Phytocompounds present in the various extracts of *Geranium wallichianum* analyzed through LC/MS technique

S. NO.	Methanol Extract	Ethanol Extract	Ethyl Acetate Extract	Petroleum ether Extract
1.	α - Bisabolol	Linalool	Ferulic acid ethyl ester	Ferulic acid methyl ester
2.	β-Elemene	Quinic acid	Germacrone	Geryl butyrate
3.	β-udesmol	Docosane	Germacrone iso	γ- cadenene
4.	Quercetin	Tridecanal	Neryl acetate	Modhephene
5.	Caffeic acid	β- Selinene	Germacrene-D	Benzaldehyde
6.	Kaempferol	Nerolidol acetate	Germacrene-A	Ishwarane
7.	Thymol	Endo-Borneol	Kaempherol	Caryophyllene oxide
8.	Camphene	Sabinene	Elatine	5,5-Dimethyl-2(5H)-furanone
9.	β-Elemene	Trans-Linalool oxide	Geranylacetone	1–Octenyl acetate
10.	Kaempferol 3,7,4'-trimethyl ether	Camphor	Cuparene	Benzyl benzoate
11.	Geraniol	p-Cymene	Herniarin	Hexanal
12.	cis-Linalool oxide	γ-Curcumene	p-Cymen-8-ol	Limonene
13.	p-Cymen-7-ol	Trans-Pinocarveol	Linalyl acetate	2,4,6-trihydroxyethylbenzoate
14.	p-Cymene	Epicatechin	Geranyl acetate	Piperitone
15.	Methyl hexadecanoate	β-Himachalene	Nerolidol acetate	Hexanoic acid
16.	p-coumaric acid	Myricetin	Gallic acid	Metronidazole
17.	Homoeriodictyol	Caftaric acid	Bornyl acetate	Quercetin-4'-O-glucoside
18.	Lutein	β-Pinene	Geraniin	Caffeic acid
19.	Kaempferol 3-methyl ether	γ-Elemene	Hexadecanal	3-Methyl-2-hexanone
20.	Kaempferol 4'-methyl ether	Nerol	Catechin	Tetrahydrogeranyl acetone
21.	Methyl tetradecanoate	Quercetin-3-O-rhamnogalactoside	Kaempferol-3-O-glucoside	Quercetin 3,3'-dimethyl ether
22.	Menthol	(E)-2-Methyl-2-butenoic acid	Quercetin 3,7, 3'-trimethyl ether	Hyperoside
23.	Ferulic acid	Cis-Jasmone	Beta Caryophyllene	Carvone
24.	Emetine	Maltol	Apigenin	Tricyclene
25.	Acetovanillone	Borneol	Quercetin-3-O-galactoside	2,6-Dimethyl-1,7-octadien-3,6-diola
26.	Dichloromethane–MeOH	Gallic acid 3,5-dimethyl ether	Gallotannin	Hyperin
27.	Kaempferol-3-O-rhamnogalactoside	Sringic acid	Morin	Benzoic acid
28.	Phytol	Chlorogenic acid	3',4'-Dimethoxyflavone	Rimantadine
29.	Apigenin	Luteolin	Ellagitannin	Quercetin-3-O-rhamnoside
30.	Oleanolic acid	Kobusin	Ethyl hexadecanoate	Eudesmin
31.	Germacrene-B	Retusin	Kaempferol-3-O-rutinoside	2,6-Dimethyl-3,7-octadiene-2,6-diol
32.	3-Methyl-2-hexanone	Cubeban-11-ol	Apigenin	(E)-Furan linalool oxide
33.	Ellagic acid	Isophytol	Beta sitosterol galactoside	
34.	3-Methyl-2-butenal		Undecanal	
35.	Homoeriodictyol		Quercetin-7-O-glucoside	
36.	Beta sitosterol		Quercetin-3-O rutinoside	
37.	Quercetin-3-O-glucoside		Lutein	
38.	Cadalene			
39.	α- Cadinol			
40.	Quercetin-3-O-galactoside			
41.	Germacrene-A			
