# Supplementary material for: Molecular docking analysis and evaluation of the antimicrobial properties of the constituents of Geranium wallichianum D. Don ex Sweet from Kashmir Himalaya
Source: Sci Rep. 2022 Jul 22;12:12547. doi: 10.1038/s41598-022-16102-9 (PMC9307801; doi:10.1038/s41598-022-16102-9)
Supplement: Supplementary file 2 — Supplementary Legends. [file 41598_2022_16102_MOESM2_ESM.docx]

**Supplementary Information**

LC–MS, Molecular docking and MD simulation files are available as s supplementary data with this manuscript.
